# Overtopping risk of high-hazard embankment dam under climate change condition

**DOI:** 10.1371/journal.pone.0311181

**Published:** 2025-02-27

**Authors:** Wan Noorul Hafilah Wan Ariffin, Lariyah Mohd Sidek, Hidayah Basri, Noorhayati Idros, M. Torres Adrian, Noor Hisham Abd Ghani, Hazri Mohd Khambali, Siti Mariam Allias Omar, Muhammad Izzat Azhar Khebir, Ali Najah Ahmed

**Affiliations:** 1 Dam Safety and Sustainability Intelligence Group, Institute of Energy Infrastructure, Universiti Tenaga Nasional (UNITEN), Selangor, Malaysia; 2 INGENIERÍA DE PRESAS S.L., Valencia, Spain; 3 National Water Research Institute of Malaysia (NAHRIM), Ministry of Energy Transition and Water Transformation (PETRA), Selangor, Malaysia; 4 Design and Dam Division, Department of Irrigation & Drainage, Kuala Lumpur, Malaysia; 5 Department of Engineering School of Engineering and Technology, Sunway University, Bandar Sunway, Petaling Jaya, Malaysia; ICIMOD: International Centre for Integrated Mountain Development, NEPAL

## Abstract

Climate change poses an escalating threat to the safety of high-hazard embankment dams, increases flood discharge impacting dam overtopping risk by altering the hydrological load of the original dam designed capacity. This paper’s primary aims are to evaluate climate change’s influence on extreme rainfall events and their impact on dam safety and to assess the overtopping risk of Batu Dam under various climate scenarios. This study focusses on assessing the overtopping risk of Batu Dam in Malaysia, utilizing regional climate model projections from the Coupled Model Intercomparison Project 5 (CMIP5) spanning 2020 to 2100. Three Representative Concentration Pathways (RCPs)—RCP4.5, RCP6.0, and RCP8.5 as the scenario and divide into 3 period of study: early century (2020–2046), mid (2047–2073) and late-century (2074–2100) evaluated with hydrological analysis to access the dam safety. Using the Linear Scaling Method (LSM), we corrected the bias projection rainfall data from three Regional Climate Models (RCMs) for the RCPs. Future Probable Maximum Precipitation (PMP) was estimated using statistical analysis techniques developed by the National Hydraulic Research Institute of Malaysia (NAHRIM). Additionally, Rainfall Intensity-Duration-Frequency (IDF) curves were updated based on climate scenarios outlined in the Hydrological Procedure 2021 and the associated Climate Change Factors. The HEC-HMS hydrological model was employed to simulate PMF and IDF for ARIs ranging from 1 to 100,000 years, providing a comprehensive analysis of risks under future climatic conditions. Across all future climate scenarios, inflow events were projected to exceed the dam design inflow, with RCP8.5 indicating the highest inflow values, particularly later in the century, highlighting probability of overtopping risks. Late-century projections show inflow for ARI 50 under RCP8.5 exceeding PMF by 20%, while mid-century RCP6.0 results indicate a 15% higher inflow for ARI 50000. Early-century RCP4.5 shows a 10% increase for ARI 100000 compared to PMF. The study advocates adaptive dam safety management and flood protection measures. This research provides crucial insights for embankment dam owners, stressing the urgent need to address Batu Dam’s vulnerability to extreme flooding amidst climate change and emphasizing proactive measures to fortify critical infrastructure and protect downstream communities.

## Introduction

Dams have been built worldwide to offer a range of benefits, including generating hydropower, controlling floods, supplying water to residential areas, and supporting agricultural activities, with the overall goal of fostering the progress of nations. There are 60,000 dams worldwide, with China and the USA, the world’s largest economies, leading by constructing the most dams [1]. As the demand for water and economic growth has increased, there has been a significant increase in the construction of large dams [2]. Embankment dams are the most built dams, with 64% being earth fill, 13% being rockfill, 23% concrete and other forms and the remaining 32% being concrete and other forms of dams. The remaining 32% are composed of concrete and other forms of dams [3]. As the oldest and most prevalent large dam type [4], embankment dams hold significant importance globally due to their cost-effectiveness and availability of material [5]. However, they also face the highest frequency of failures among dams [1].

Apart from the numerous benefits provided by dams, they also represent critical infrastructure, and in the event of failure, downstream areas can suffer catastrophic consequences [6]. The failure of embankment dam is mainly driven by hydrological overload [7]. Generally, an embankment dam is constructed using compacted earth, rocks, or other suitable material and not designed to handle overtopping, as that can cause rapid erosion of embankment dam material. The pressure put by climate change on dams is beyond the dam’s original design. In the past, many dams were developed assuming stationary climate conditions [8]. Today, resilient, and sustainable water planning and management must consider global climate change, which determines the region’s socioeconomic growth [9]

Climate change has caused a rise in air temperatures, resulting in a 7% increase in the atmosphere’s ability to hold water for every 1°C increase. This has led to more frequent and intense rainfall events [9]. The past three decades saw the warmest years in history, with global temperatures rising between 0.65 and 1.06°C from 1880 to 2012 [10]. There have been alterations in rainfall patterns, resulting in a higher occurrence of intense rainfall in certain areas [11, 12], whereas other regions have experienced a decrease in rainfall [13, 14]. Studying the trend of rainfall at the regional level is highly crucial for precisely modelling how rainwater responds in a given area. Several studies have explored how climate change affects Malaysia’s hydrological pattern, focusing on exceptionally heavy rainfall. [15] examined rainfall-related extremes throughout Malaysia’s east coast from 1971 to 2010, while [16] examined precipitation events from 1975 to 2004.

In 2015, another study studied Malaysian seasonal extreme precipitation from 1975 to 2010. [17] found more Northeast monsoon wet days and rainfall depth. [18] showed inter-monsoon 1-hour storm intensity above 20 mm increasing. [19] found an increase in daily and yearly rainfall depths along Peninsular Malaysia’s east coast. [15] studied the impact of El Niño and La Niña on rainfall extremes in Malaysia. These studies provide evidence of Malaysia’s shifting hydrological patterns, specifically the increased frequency, severity, and length of extreme rainfall events, underlining the impact of climate change.

However, research into future hydroclimate in Malaysia is limited in the literature. [20] measured Peninsular Malaysia’s water resources from 2025 to 2050. Then in 2013, with the input Global Climate Change Model (GCM) model, research was done on rainfall-related extremes along Malaysia’s east coast between 1971 and 2010 compared to the observed period. [21] conducted an assessment of the hydrological conditions that are expected to occur in the future over the Muda and Dungun watersheds in Peninsular Malaysia. In conclusion, every study demonstrates that extreme precipitation patterns in Malaysia are on the rise. In 2019, a study on the effects of climate change on precipitation, temperature, and soil moisture storage was conducted [22]. In conclusion, all studies show rising trends of extreme precipitation in Malaysia.

Climate change is expected to affect the hydrological regime and the volume of water flowing into reservoirs [23]. Climate change is expected to increase the frequency of heavy rainfall events, which will affect the factors contributing to dam failure [24]. The United States experiences an average of 10 dam failures per year, with one of the most severe events being the South Fork Dam disaster induced by significant rainfall [25]. This highlights the global significance of this issue. Malaysia has also experienced seven dam-related accidents, all attributed to hydrological factors. Notable incidents include the 1970 Panchor Dam failure in Penang, caused by intense rain-induced erosion of upstream slopes, and the 1981 collapse of the Batu Arang Dam in Selangor due to prolonged rains. The Anak Endau Dam in Johor also collapsed in 1986, and a tragic incident occurred at the Sultan Abu Bakar Hydro Power Dam in Pahang in October 2013, claiming four lives in a water overflow incident [26]. More recently, in March 2021, a flood incident at the Kenyir Dam area in Terengganu caused damage to the flora and fauna [27]. Even if the likelihood of a dam failure is low, the consequences of a failure are enormous, with severe consequences for life, the community, the economy, and the environment [28]. Dams globally face increased threats of overtopping due to additional loads exceeding their original design capacity. Overtopping is a major concern to dam safety around the world, particularly for embankment dams, because it is the leading cause of dam collapses, which can have disastrous repercussions such as downstream floods and loss of life [29].

Assessing the potential risk of overtopping in embankment dams is crucial given the magnitude of the anticipated rise in extreme precipitation events caused by climate change [30]. The occurrence of more frequent and severe floods as a result of climate change in Malaysia poses an increased danger of dam overtopping [27]. This, in turn, might lead to dam failure and raise safety issues for stakeholders involved in water infrastructure [31]. Research has demonstrated that phenomena like variations in water levels and instances of overflowing during heavy rain can have a substantial effect on the collapse of embankments and their tendency to be overtopped [32]. The current global standards for dam safety mainly deploy statistical or empirical methods, highlighting the importance of better understanding and enhanced safety assessments in response to hazards induced by climate change [10].

The Intergovernmental Panel on Climate Change (IPCC)’s AR5 Synthesis Report: Climate Change 2014 [10] predicts that the global temperature will surpass 1.5°C in all RCP scenarios, except RCP2.6, by the end of the 21st century. The IPCC regards RCP 4.5 as a moderate scenario, as emissions reach their maximum in approximately 2040 and then decrease. Nevertheless, it is anticipated that RCP6.0 and RCP8.5 will exceed 2°C. For instance, in Iran’s Gharesou Basin, climate projections for the period 2040–2069 indicate temperature increases and precipitation changes of ±30%, resulting in runoff variations ranging from -90% to 120% [33]. Similarly, in Sri Lanka’s Nilwala River Basin, future annual streamflow is expected to rise by 59.30% and 65.79% under RCP4.5 and RCP8.5 scenarios, respectively [34]. These projections underscore the heightened risk of overtopping, which can have catastrophic consequences for high-hazard embankment dams. Furthermore, a case study of the Denawaka Ganga mini-hydropower plant in Sri Lanka highlighted some environmental concerns but also significant positive social impacts [32]. Climate change is anticipated to substantially affect water resources and dams across various regions, necessitating comprehensive assessments using hydrological models.

This study integrated regional climate models with hydrological simulations to predict future scenarios and assess their impacts on Batu Dam, a high-hazard dam located in Selangor. The objective of this study is to assess the overtopping risk of embankment dams caused by hydrological load under the impact of climate change in the twenty-first century. The analysis has been described under a hydrological scenario, where flooding is the main load on which dams are imposed. The climate change projection data was obtained by downscaling Global Climate Models (GCM) to regional data under Coupled Model Intercomparison Project 5 (CMIP5) by NAHRIM spanning the years 2020 to 2100. The projected changes in future rainfall pattern associated with climate change, and the reliability of existing IDF curves generated from historical data and simplified assumptions, need to be reviewed. Adjustments to the current IDF curves are necessary for reliable projections of future rainfall under different climate change scenarios. This study used generated GCM rainfall data simulations for four stations in the catchment area, considering RCP4.5, RCP6.0, and RCP8.5 scenarios. Then, an analysis of PMP is conducted by the NAHRIM envelope curve [22], which will then be an input to estimate the PMF. The result of simulated inflow design flood and check with the dam designed inflow to predict the probability of overtopping.

The overtopping risk of high-hazard embankment dams is a critical concern, especially under the changing climate conditions in Malaysia. As global temperatures rise and weather patterns become more unpredictable, the potential for extreme rainfall events leading to dam overtopping risk increases. Understanding and mitigating this risk is essential for ensuring the safety and stability of these vital structures. Dam safety management must consider climate change impacts [35]. Addressing this risk is imperative to safeguard communities and infrastructure downstream of these dams. Besides, to further carry out the adaptation of climate change under Sustainable Development Goals (SDG) 13 toward climate action for global sustainability.

### Study area

**Fig 1:**
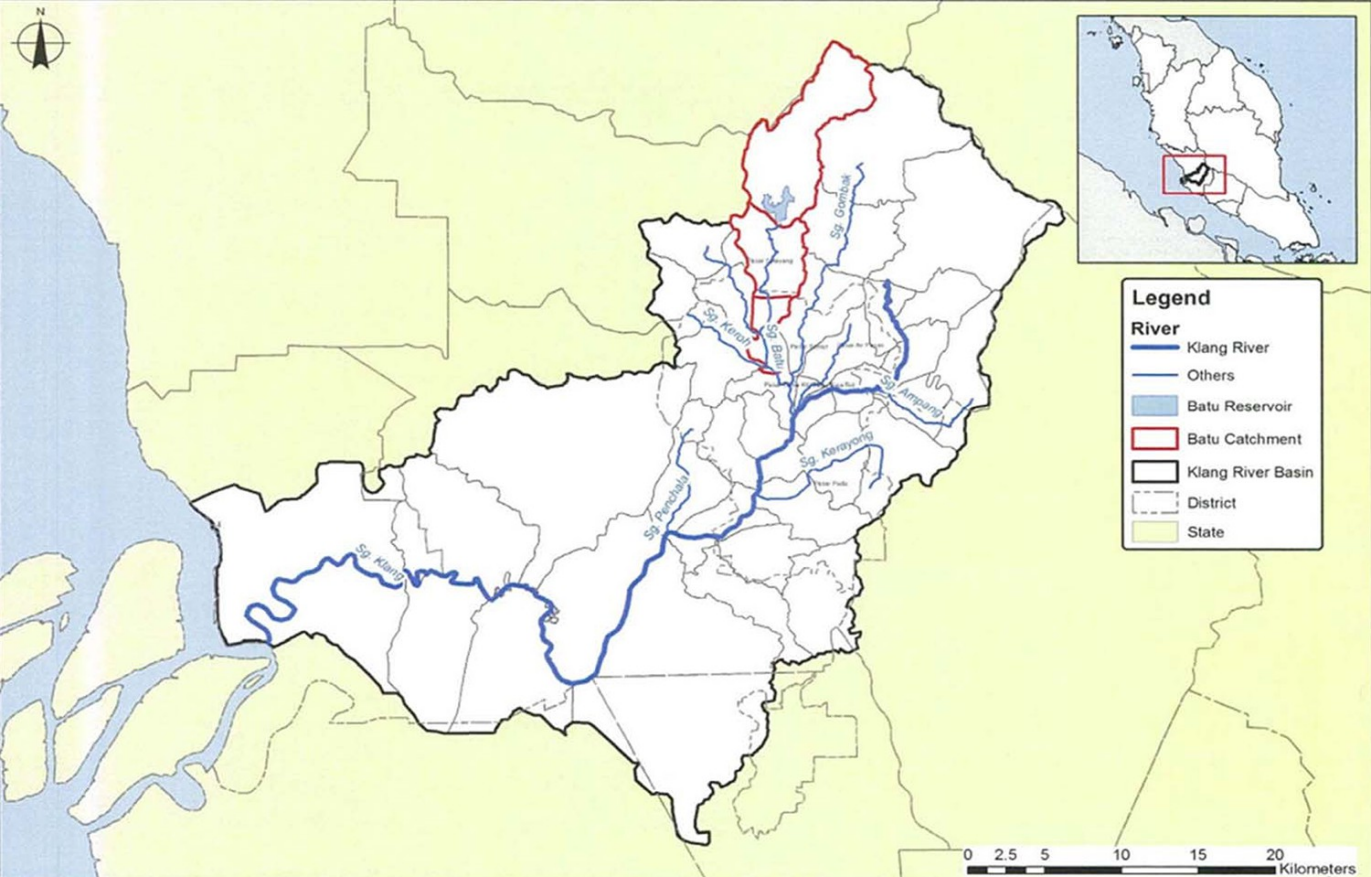
Study area, Batu Dam, Selangor. Batu Dam located at upstream Klang River legend in Blue Line, and located at upper part of Sg Batu Catchment in Red Line, The Batu Dam reservoir area filled with Blue.

The Batu Dam is considered the most critical dam due to its high hazard rating due to its highest crest elevation, water level, and shortest distance to the downstream populous region [17]. The downstream region of the dam is densely populated and has been constructed to safeguard almost 1.25 million people affected by the threat of flooding. It encompasses a range of industries, treatment plants for water, and residential areas. Due to a major flood in Kuala Lumpur in 1971, the establishment of this dam was mandated as part of a larger project to minimise the occurrence of future flooding mainly, protect against flooding in the Kuala Lumpur metropolitan area, and it also serves as a water supply reservoir. A surcharge, collectively with a spillway and outlet, is established to allow for a discharge of 287.4 m3/s. This is done to defend from the inflow design flood. Fig 1 shows the study area of Batu Dam.

### Data

**Fig 2:**
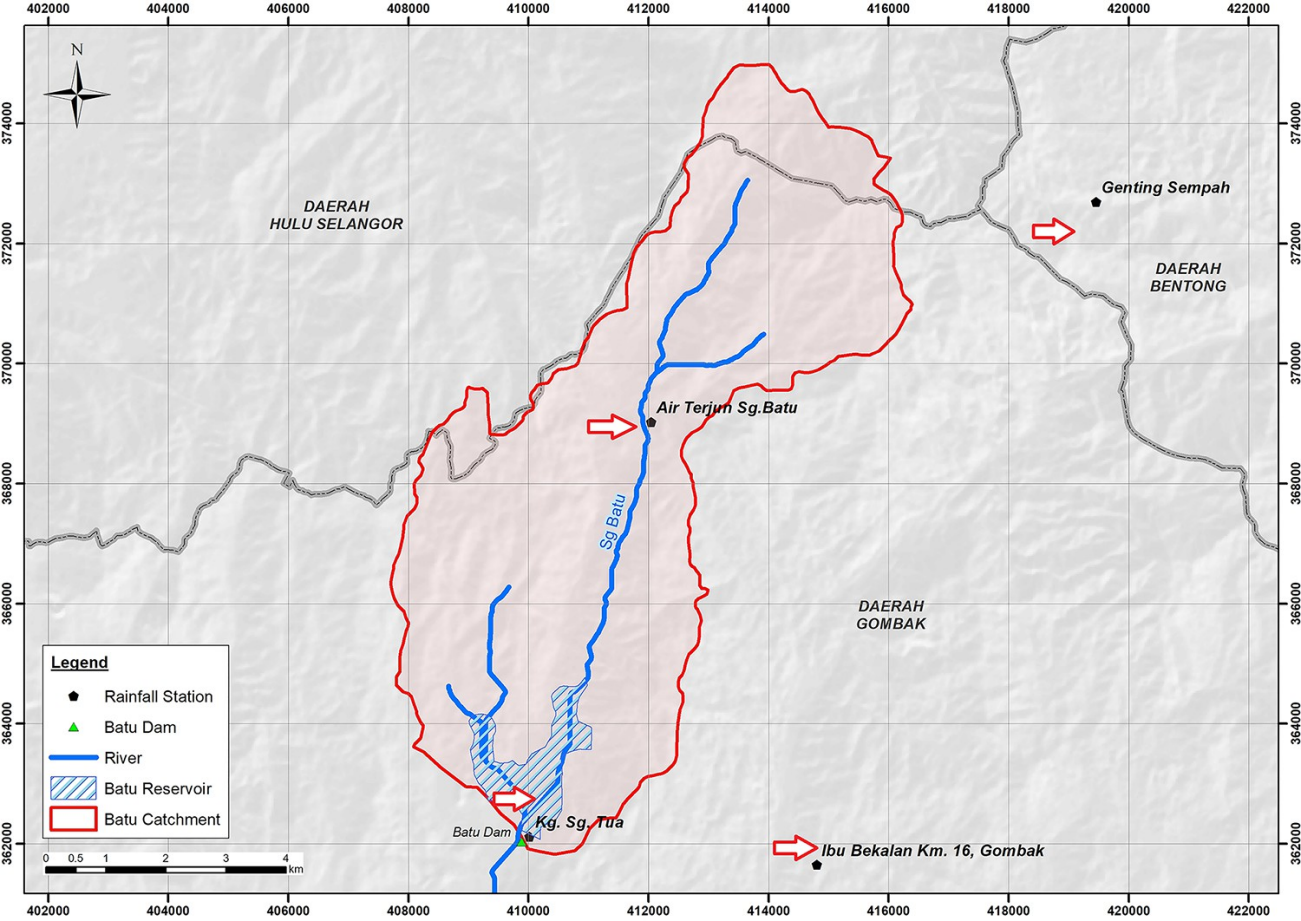
Shows the Batu Dam catchment boundary and rainfall station locations. 2 station: Station Kg Sg Tua and Station Air Terjun located within the Batu Dam catchment, while another 2 station Station Genting Sempah and Station Ibu Bekalan KM 16 Gombak located outside the Batu Dam Catchment.

This study obtained historical hydrological data from Malaysia Department of Irrigation and Drainage **(DID)** for four rainfall stations near the Batu Dam basin as illustrated in Fig 2. These stations include (i) 3216001—Kampung Sungai Tua (Kg. Sg. Tua), (ii) 3217001—Ibu Bekalan KM16, (iii) 3317001—Air Terjun, and (iv) 3317004 –Genting Sempah as show in Fig 2. The dataset’s gaps were filled using the Close Neighbour approach. Climate change’s impact on future water resources has been projected using GCM simulation data. Regional Climate Models (RCMs) are regional climate projections and climatic consequences based on downscaled GCM simulations. Table 1 summarizes the 16 climate model by driving GCM provided by the Malaysian National Water Research Organisation (NAHRIM) for this study.

**Table 1:** Global Climate Models (GCM) for the 16 future projections from 2006 to 2100. (NAHRIM, 2021). The GCM that have all the scenario in RCP2.5, RCP4.5 and RCP6.0 and RCP8.0 are highlighted in yellow.

GCM	Historical	RCP2.6	RCP4.5	RCP6.0	RCP8.5
CCSM4	x	-	x	-	x
MIROC5	x	x	x	x	x
MRI-CGCM3	x	-	x	-	x
GFDL-ESM2M	x	x	x	x	x
IPSL-CM5A-LR	x	x	x	x	x

## Methods

Methodologies developed for assessing the safety of embankment dams in the context of climate change. The objective is to assess the risk of overtopping of the dam. In the context of dam safety, the risk is the combination of three elements: the possibility of impact (dam failure), the likelihood of the event (failure probability), and the potential consequences (effects/failure consequences) [36]. Eq (1) can be employed for estimating risk [36]. Risk can be calculated using Eq (1):

R=∑ep(e)∙p(f|e)∙C(f|e) (1)

**Fig 3:**
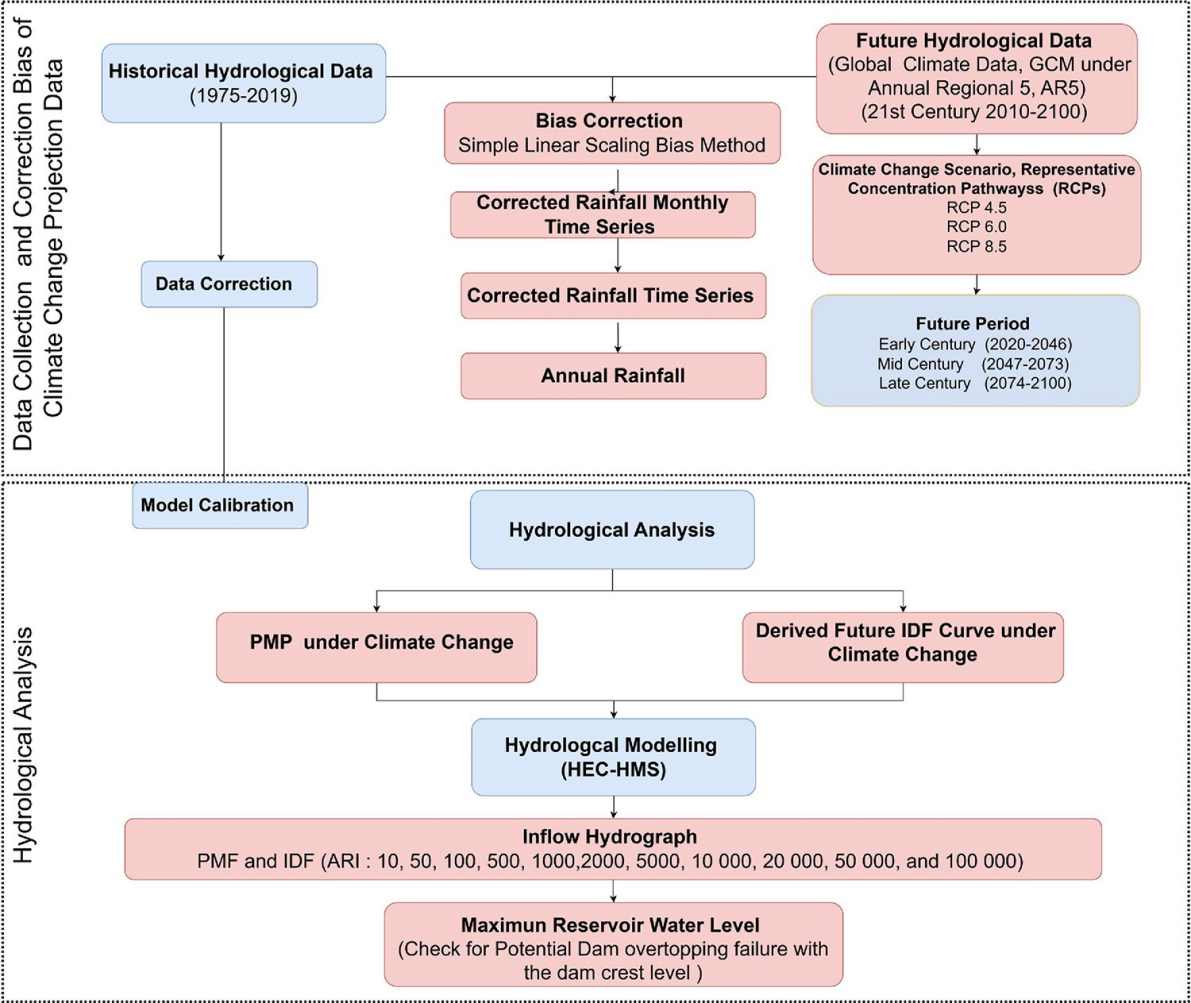
Flowchart of the methodology to assess the overtopping risk due to climate change. The flow chart devided by 2 part, first part on data collection of historical data and projection climate change data, corrected by biased analysis and second part on hydrological analysis.

If the risk is expresses in term of social or economic consequences per year, the summation is determined for all event of an incident, *e*, where *p(e)* is the likelihood of an incident, Here, p(e) represents the probability of the incident happening, p(f|e) represents the probability of failure resulting from the incident e, and C(f|e) represents the consequences resulting from the risk Climate change is expected to increase the frequency of heavy rainfall events, which will affect the factors contributing to dam failure [24]. These effects are expected to change over time. The climate projections for the end of the twenty-first century will be different from those for the historical period, and therefore may be subject to change. Fig 3 outlines the methodology for applying the steps based on the available hydroclimate observations for historical climate projections. This methodology aims to assess the impact of climate change on the risk of embankment dam safety due to overtopping.

### Bias correction of climate projections

Data for future projected rainfall is corrected using the Linear Scaling bias correction method as shown in Eq (2); it was the best-performing correction method for the correction of long-term trends [12]. This method is mainly used to correct errors to improve the accuracy of climate change data by offering a simple and flexible approach to correcting model biases. Additionally, it helps to reduce uncertainty by improving the reliability of climate change predictions. Specifically, the linear scaling method adjusts monthly mean values resulting in corrected data that closely aligns with the original GCM data.

$$CF=\frac{\left(\bar{x}o\right)}{\left(\bar{x}p\right)}$$ (2)

Where CF is Correction Factor, $\bar{x}o$ is Mean of monthly observed data, $\bar{x}p$ is mean of monthly projected data, and Corrected Projected Data is Raw Projected Data × Correction Factor (CF) [37].This procedure is independently applied to each climate forecast data under three future Periods (1, 2, and 3), with 2010–2020 data calibrating for the correction component.

### Ensemble approach

To address the inherent uncertainty in future climate models we adopted an ensemble approach by combining outputs from the selected three Global Climate Models (GCMs): MIROC5, GFDL-ESM2M, and IPSL-CM5A-LR at three different Representative Concentration Pathways (RCPs) scenarios: 4.5, 6.0, and 8.5. Ensemble approaches using multiple GCMs are widely employed to address uncertainties in climate change projections and their impacts on flood risk [22, 30, 31]. This method is particularly pertinent for evaluating extreme values in risk assessments [22], which are crucial for assessing overtopping risks and flood scenarios.

The ensemble approach enables us to capture a range of potential future climate scenarios by integrating outputs from different GCMs. Each GCM may vary in its sensitivity to various climate drivers, resulting in different projections of future climate conditions. By using an ensemble of GCMs, we can reduce the uncertainties associated with individual models and provide a more comprehensive understanding of potential future climates.

By using this ensemble approach, our study provides a comprehensive assessment of the overtopping risks and flood scenarios under future climate conditions, addressing the uncertainties inherent in climate change projections.

### Hydrological analysis

#### Probable Maximum Precipitation (PMP)

PMP refers to the maximum rainfall that can accumulate in a given catchment area within a specified time and location [38] PMP is frequently used in the calculation of the Probable Maximum Flood (PMF) [8]. It is found that the NAHRIM Km envelope curve is more reliable compared to Hershfied method because it provides a more precise PMP estimate [39]. Thus, considering the worst-case scenario, the NAHRIM Km Envelope Curve approach was used to develop a frequency equation for PMP precipitation. It is often used for rainfall stations that have extensive records [40]. The PMP is calculated for different time periods, including hourly (1, 3, 6, and 12 hours) and daily (1, 2, 3, and 5 days).

#### Historical rainfall intensity duration frequency (IDF) curve

The IDF relationship for a given return period is a special case of the generalized formula in Eq (3). In this equation, ω, v, θ, and η are positive coefficients, with the condition that vη ≤ 1. However, if we assume that v = 1, the resulting error is significantly lower than the regular estimation error de-scribed in Eq (4).


$$Intensity=\frac{\omega }{(d^{v}+{\theta )}^{\eta }}$$ (3)

$$Intensity=\frac{\omega }{(d+{\theta )}^{\eta }}$$ (4)

For any two return periods T1 and T2, where T1 < T2 yield a set of limitation in Eq 4 in which θ1 = θ2 = θ ≥ 0, 0 < η1 = η2 = η < 1, and ω1 > ω2> 0. ω is considered to be an increasing function of the return period T. This leads to a generalised IDF relationship, as shown in Eq 5, which captures the dependence of I on T and d. In this function, b(d) = (d+θ) η where θ and η are the parameters to be estimated (θ>0, 0<η<1).


$$i=\frac{a(T)}{b(d)}$$ (5)

The function a(T) is determined by the probability distribution function of the maximum rainfall in-tensity, I(d). In this equation, the intensity I(d) represents a specific time with a distribution FI(d)(i;d), resulting in a variable distribution X≈I(d)b(d) that closely approximates the intensity re-scaled by b(d). Mathematically, this can be expressed as Eq 6.


$$X_{T}=a(T)=F_{Y}^{-1}(1-\frac{1}{T})$$ (6)

To simplify the parameter of the GEV function, a(T), can be represented using: Eq 7

$$a(T)=\lambda T^{k}$$ (7)

and finally, Eq 8 was transformed from Eq 5 and Eq 8 in general term as:

$$i_{Historical}=\frac{\lambda T^{k}}{{\left(d+\theta \right)}^{\eta }}$$ (8)

#### Development of future IDF curve

The ongoing trend of global warming makes it imperative to recognize that the current IDF curves, which are exclusively based on historical data, may not be sufficient for precisely projecting future patterns of rainfall [41]. The future IDF curves were developed using observed data and bias-corrected simulated datasets obtained from the climate change model used to simulate future climate conditions.

A climate change factor (CCF) was calculated and applied to the IDF curves to account for the impact of climate change. The CCF allows for estimating new rainfall intensities that consider the effects of climate change. This adjustment helps account for the potential changes in precipitation patterns and intensities expected in the future due to climate change.

By incorporating the CCF into the IDF curves, the study aimed to provide estimates of future rainfall intensities that account for the anticipated climate change effects. These estimates are essential for designing resilient infrastructure and making informed flood management and water resource planning decisions. The IDF curve develops for return periods of 5, 10, 20, 50, 100, 500, and 1000, 5000, 10,000, 20,000, 50,000, and 100,000 years at Annual Maximum Series (AMS) moving totals for various durations of 1, 2, 3, 4, and 5 hours. AMS moving totals help capture the extreme precipitation events over consecutive hours within a year.

Future IDF Curve obtained by derived Climate Change Factor (CCF). The following Eq (9) describes the general relationship between IDF future projections:

$$I_{F}=\frac{\lambda^{\prime }T^{k}}{{\left(d+\theta \right)}^{\eta }}$$ (9)

Where, rainfall intensity for the future. The original was modified, incorporating CCF. Then, the future can be written as: Where, ***I_F_*** Rainfall intensity for future. The original λ was modified as λ^’, which incorporates CCF. Then, the future λ^’ can be written as:

$$\lambda^{\prime }=CCF_{T}$$ (10)

Thus, the future design rainfall:

$$I_{F}=CCF_{T}*\frac{\lambda T^{k}}{{\left(d+\theta \right)}^{\eta }}$$ (11)

Then, the annual maximum of 1-hr rainfall events from climate models was used to calculate the CCF and subsequently applied to the current curve to develop the future IDF curves.

### Hydrological model

In this study, the US Army Corps of Engineers Hydrologic Engineering Centre (USACE) used their Hydrologic Modelling System (HEC-HMS) software to simulate flow in branched watershed systems and determine the inflows across a wide range of return periods (1 to 100,000 years) and simulating the maximum possible flood (PMF) scenarios under various climate change conditions for Batu Dam.

**Fig 4:**
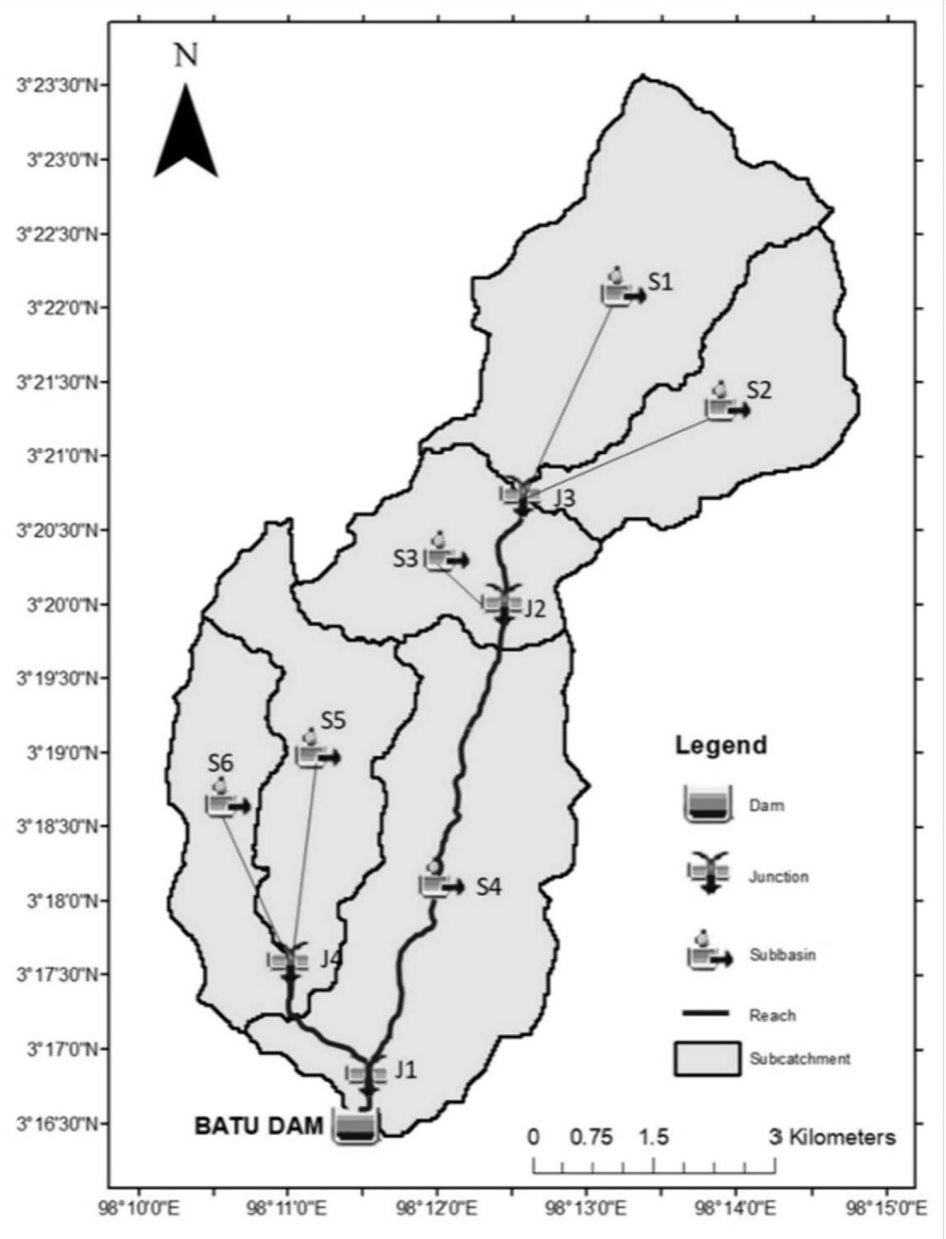
Subareas basin model setup. The dam catchment divided into 6 sub catchments coded by S1 to S6.

1. **Dividing Drainage Area into Subareas:** The 50 km^2^ Batu Dam catchment is divided into six sub-catchments (S1-S6) as in Fig 4 Subareas basin model setup based on different soil types and land use patterns at Batu Dam. This is achieved using GIS tools within HEC-HMS, with a Digital Elevation Model (DEM) from ALOS PALSAR (30 m resolution) in the Universal Transverse Mercator (UTM) coordinate system.
2. **Determination of Catchment’s Physical Characteristics:** The physical characteristics, such as area, stream length, and slope, are determined using Global Mapper or GIS tools. These characteristics include the longest path of the stream, the centroid length (Lc), and elevation profiles. Data from the Revised Inflow Design Flood Batu Dam Kuala Lumpur Flood Mitigation Project Study (USBR, 1984) is also utilized. Detailed information on the physical characteristics is shown in Table 2.
3. **Input parameters for HEC-HMS:** All physical characteristics of the Batu Dam sub catchments such as the area of sub-catchments, the longest flow path, elevation, and slope of the river can be obtained from the GIS features in HEC-HMS model after completing the delineation process, while study by the authors of [27] still used the ArcGIS tool to determine the physical parameter of the catchment for the input in the HEC-HMS model. All these physical characteristics then will be used to determine the input parameters by using the Malaysia guideline from [23]and calibrated by using guidelines from [14] as tabulated in Table 3.

**Table 2:** Batu Dam catchment details. The input for each sub catchment; River Name, Area, Length, Lag and Lc (Length at centroid).

Catchment	Sub-Catchments	River Name	Area (km2)	Length (km)	Lag (hr)	Lc (length at centroid, km)
**Batu Dam**	SC1A	Sg Batu	11.35	5.26	2.71	2.63
	SC1B	Sg Batu	7.32	5.07	2.64	2.54
	SC1C	Sg Batu	14.66	6.35	3.35	2.87
	SC2	Sg Tua	4.61	3.11	2.12	2.3
	SC3	Sg Bisul	4.11	4.12	2.82	2.06
	SC4		2.39			
	SC5		1.29			
	SC6		0.8			
	**BATU RESERVOIR**		2.13			
	**TOTAL**		**51.3**	**26.56**		

**Table 3:** Physical parameters for study area. The parameter input of loss method, transform method and based flow based on sub-basin.

Sub-Basin	Loss Method			Transform Method		Baseflow Method
	Initial and Constant			Clark (UH)		Constant Monthly
	Initial Loss (mm)	Constant Rate (mm/h)	Imperviousness (%)	SCS Tc Eq. (h)	R (h) HP 27	HP27 (m^3^/s)
S1	30	2.5	10	3.110	1.218	0.905
S2	30	2.5	10	2.660	1.116	0.584
S3	30	2.5	10	2.180	0.839	0.496
S4	30	2.5	10	6.030	2.262	1.073
S5	30	2.5	10	4.680	1.957	0.597
S6	30	2.5	10	3.000	1.279	0.449

## Results and discussion

### Bias correction of the projections data

**Fig 5:**
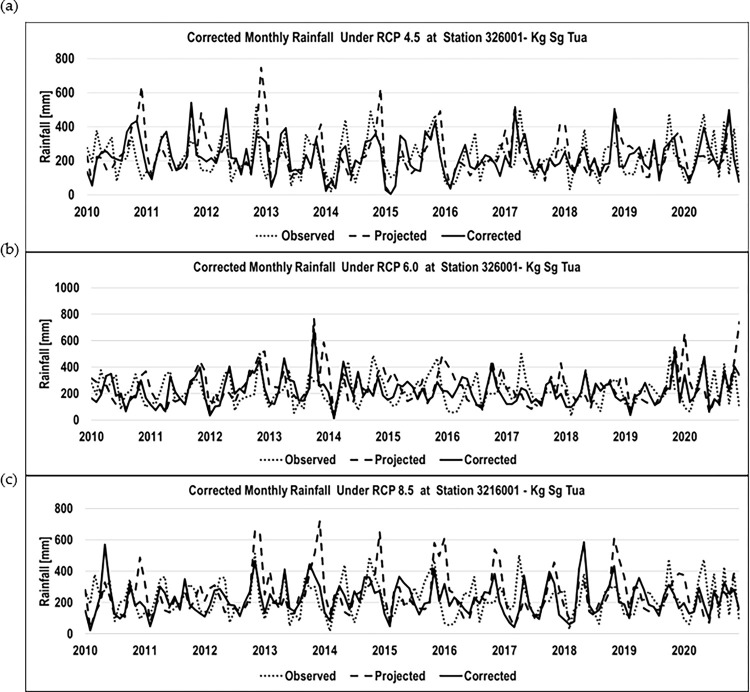
Historical monthly observed, projected, and corrected rainfall (mm). a) Corrected monthly rainfall under RCP4.5 for Station Kg Sg Tua b) Corrected monthly rainfall under RCP6.0 for Station Kg Sg Tua c) Corrected monthly rainfall under RCP 8.5 for Kg Sg Tua.

The selected station’s projected rainfall data was corrected using the Linear Scaling bias. The purpose of this correction is to enhance the accuracy and reliability of the data for a specific location or station. For this study, the projected rainfall data was corrected using observed and projected data from 2010–2020 as the calibration timeframe for the correction function. Fig 5 shows the corrected monthly rainfall, which is a combination of observational and projected data for Kg. Sg. Tua from 2010–2020 (for all RCP values. The correction factor was used to correct the projected data using Eq (2).

**Fig 6:**
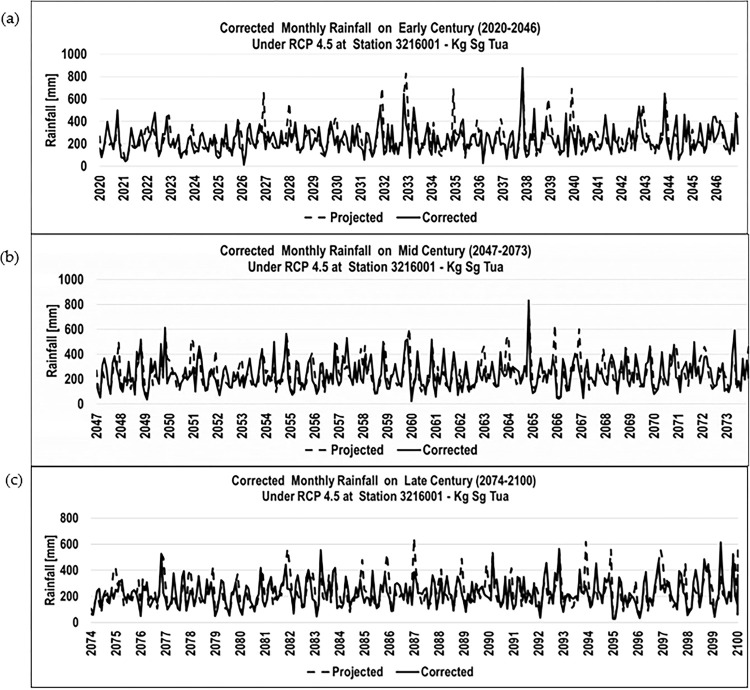
Monthly corrected projected rainfall (mm) for Kg. Sg. Tua station. a) Corrected monthly rainfall under RCP4.5 for Station Kg Sg Tua at Early Century b) Corrected monthly rainfall under RCP4.5 for Station Kg Sg Tua at Mid Century c) Corrected monthly rainfall under RCP 4.5 for Kg Sg at Late Century.

The result of the corrected rainfall data was then applied to the corrected time series (early, mid, and late centuries) for further analysis. Fig 6 illustrates the monthly corrected projected rainfall for station Kg. Sg. Tua under RCP 4.5.

### Corrected rainfall time series

**Fig 7:**
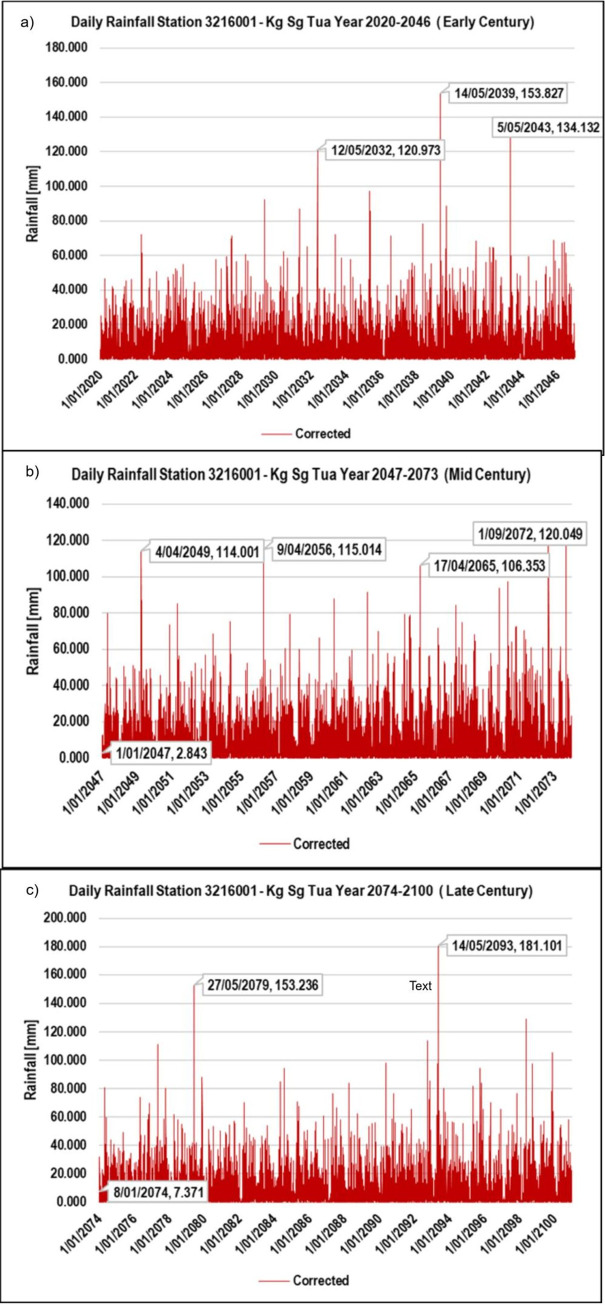
Corrected daily projected rainfall (RCP 8.5) time series for Kg. Sg. Tua rainfall station. a) Corrected daily rainfall at Early Century b) Corrected daily rainfall at Mid Century c) Corrected daily rainfall at Late Century.

As demonstrated earlier, the corrected monthly rainfall time series showed the rainfall pattern for three future study periods. Fig 7 demonstrates an example of the corrected daily projected rainfall (at RCP 8.5) for Kg. Sg. Tua rainfall station. It displays that the prediction of the two highest rainfalls will happen in the same month of May 2035 and November in 2037 in the early century, November 2060, and May 2073 (mid-century), and May 2078 and November 2081 (late century). In November and May, two distinct monsoon seasons, typically occur, northeast and southwest, respectively. In future, it shows changes in monsoon trends with decreased rainfall in the northeast monsoon while the increasing pattern in southwest monsoon [26].

**Fig 8:**
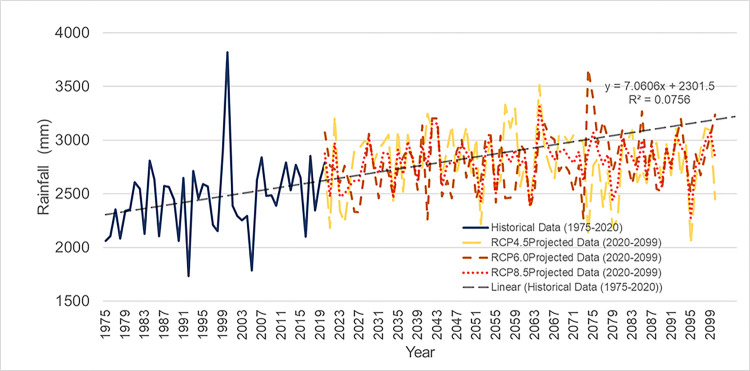
Average annual historical dan projected future rainfall for the Batu Dam catchment under all RCPs. Historical Data (1975–2020): Solid blue line shows recorded rainfall, fluctuating significantly with a spike around 1998–1999). RCP4.5 Projected Data (2020–2099): Dashed yellow line represents considerable variation in future rainfall projections. RCP6.0 Projected Data (2020–2099): Dashed orange line, showing increased variability and more extreme rainfall events. RCP8.5 Projected Data (2020–2099): Dashed red line predicting greater variability and extreme rainfall projections.

Fig 8 presents significant information into historical and future patterns of rainfall. The average yearly rainfall for the historical period was 2467 mm. All RCP scenarios, however, experienced a notable rise of 12.43% in the first part of the century, with RCP 4.5 recording the greatest average annual rainfall of 2828 mm. With values of 2887 mm and 2755 mm, respectively, into the middle of the century, RCP 4.5 and RCP 6.0 both continued to show higher average annual rainfall than in the early century. In terms of rainfall, RCP 4.5 continued to lead. However, average annual rainfall for RCP 4.5 decreased to 2715 mm in the latter part of the century, whereas average annual rainfall for RCP 6.0 (2925 mm) and RCP 8.5 (2756 mm) increased. Of the two, RCP 6.0 had the largest increase, exceeding 18.6% above the base annual rainfall. These results emphasise how critical it is to comprehend and mitigate climate change, especially in light of the many pathways of greenhouse gas concentrations that the RCP scenarios reflect.

### Hydrology analysis

#### Probable Maximum Precipitation (PMP)

**Fig 9:**
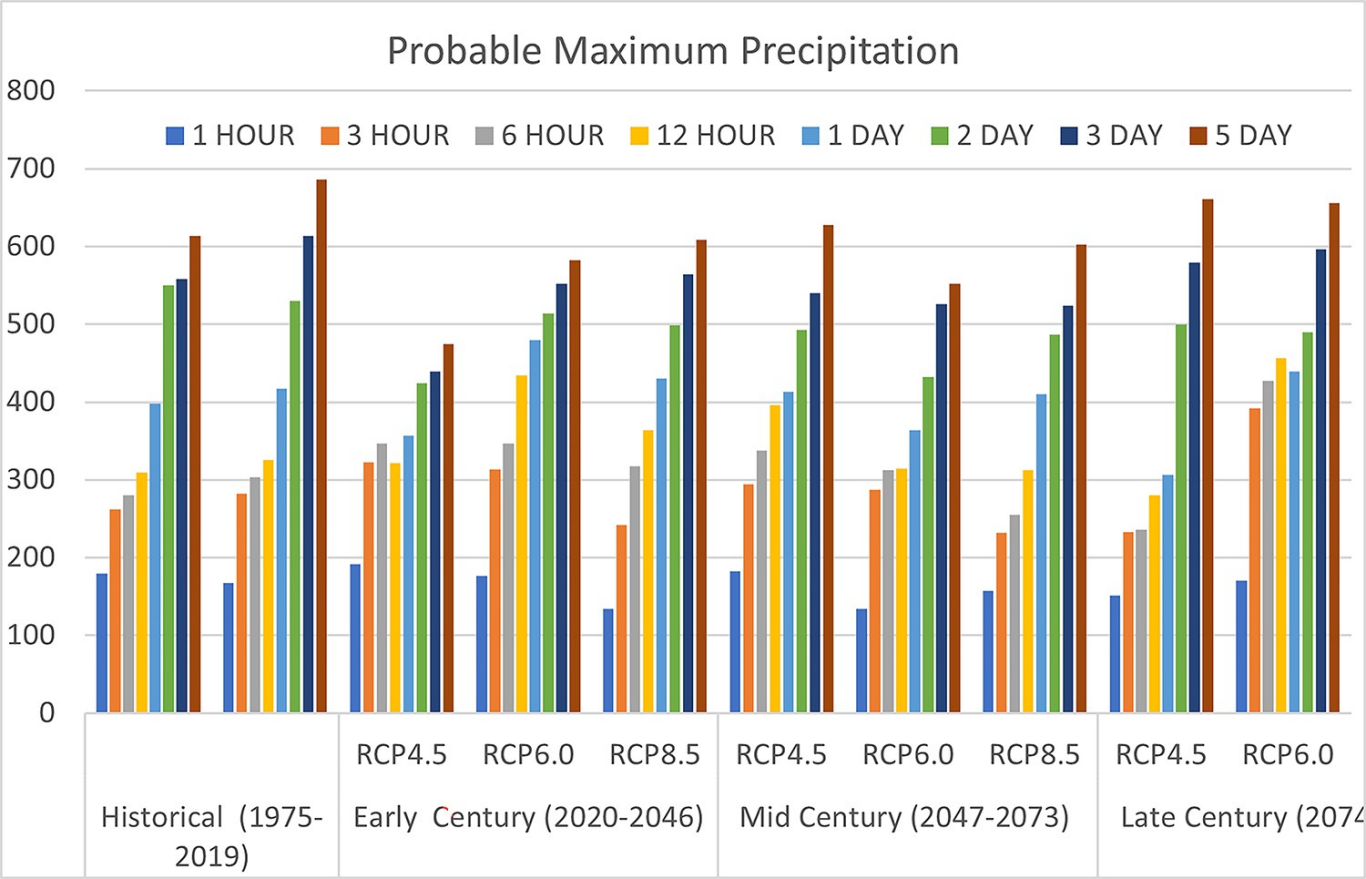
Probable maximum precipitation estimation of Batu Dam. Historical (1975–2019), Lower probable maximum precipitation compared to future scenarios. Early Century (2020–2046) RCP4.5 Early Century with a significant rise in 5-day precipitation. RCP6.0 Early Century Higher emissions result in larger increases in precipitation across all durations, RCP8.5 Early Century: The most severe emissions scenario shows a substantial rise in probable maximum precipitation, especially for 5-day events. Mid Century (2047–2073). RCP4.5 Mid Century: steady increases in rainfall. RCP6.0 Mid Century: Higher emissions continue to drive escalating rainfall intensities, particularly for 3- and 5-day events. RCP8.5 Mid Century: significant increases in probable maximum precipitation, with more frequent extreme rainfall events. Late Century (2074–2099),RCP4.5 Late Century: substantial increases in long-duration rainfall, especially 3- and 5-day events. RCP6.0 Late Century: increasing, with extreme 5-day events becoming more intense.

Probable Maximum Precipitation (PMP) was predicted for (1, 3, 6, and 12-hour) and (1, 3 and 5-day) as displayed in Fig 9. The bar chart presents the Probable Maximum Precipitation (PMP) for various durations under different climate scenarios across three time periods: Early Century (2020–2046), Mid Century (2047–2073), and Late Century (2074–2100). The climate scenarios considered are Representative Concentration Pathways (RCP) 4.5, 6.0, and 8.5. The historical data from 1975–2019 is used as a baseline for comparison, across the 21st century. In the early century, under RCP4.5, RCP6.0, and RCP8.5 scenarios, there is a noticeable increase in PMP values compared to the historical data. RCP4.5 shows the highest PMP value at 686.4 mm over five days of rainfall. However, in the mid-century, RCP6.0 surpasses RCP4.5 and RCP8.5, reaching the peak PMP value of 627.27 mm.

Moving towards the late century, RCP6.0 again projects the highest PMP value of 660.53 mm, while RCP8.5 closely follows with 655.55 mm over a 5-day event. Interestingly, RCP4.5 exhibits a declining trend in PMP values as the century progresses, while RCP6.0 and RCP8.5 show an increasing trend towards the end of the 21st century. These trends offer valuable insights into the potential changes in extreme precipitation events throughout the century, emphasizing the role of varying greenhouse gas concentration trajectories and their associated climate scenarios represented by the RCPs.

### Intensity duration frequency curve

#### Historical rainfall intensity duration frequency (IDF) curve

**Fig 10:**
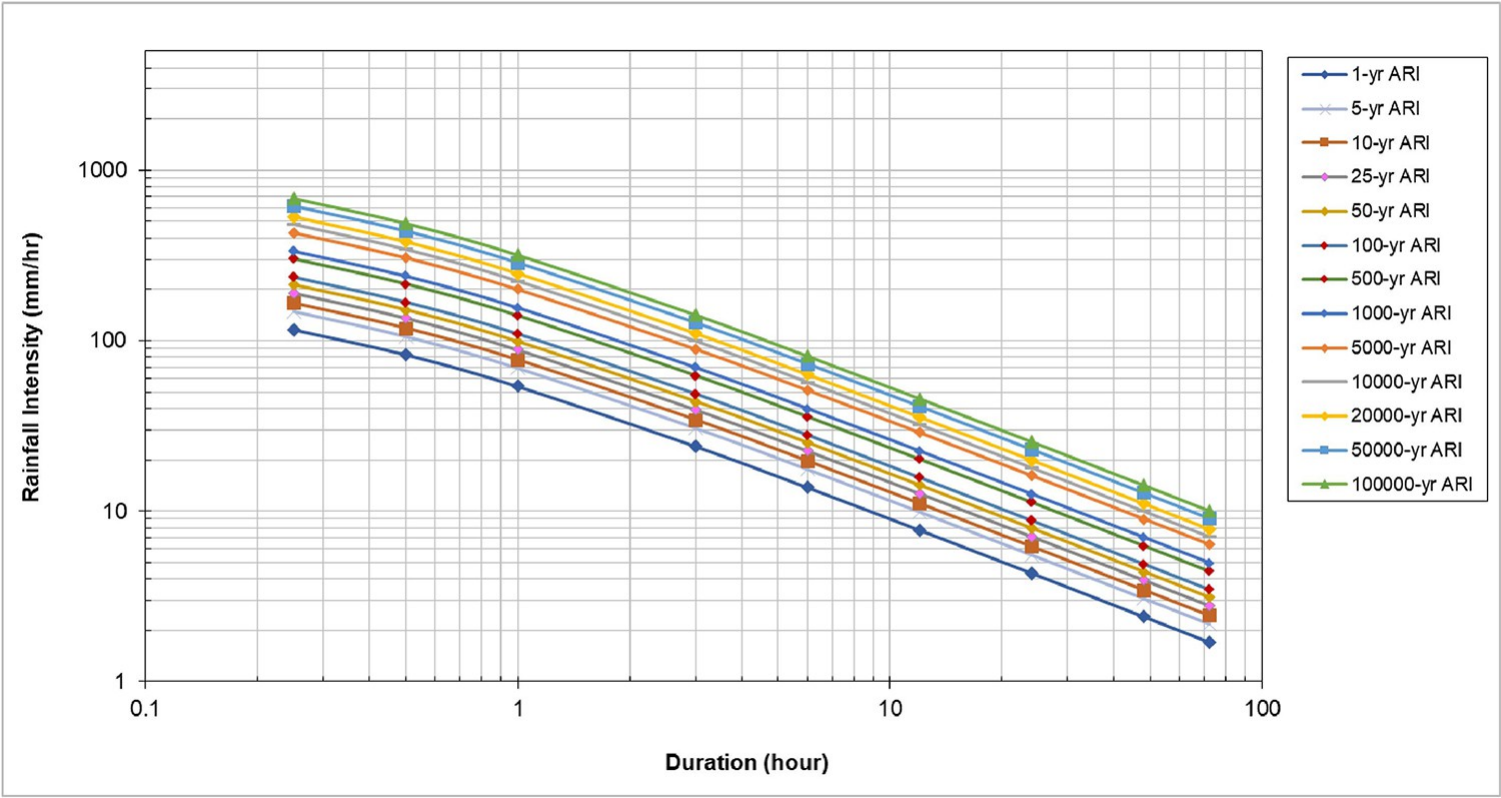
IDF curves for Batu catchment for historical data. The graph shows rainfall intensity (mm/hr) on the y-axis and duration (hours) on the x-axis, using a logarithmic scale for both axes. It displays rainfall intensity curves for different Average Recurrence Intervals (ARI), ranging from 1 year to 100,000 years.

Eq 8 was used to correct the IDF relationship for historical rainfall (Fig 10). The parameters λ, k, θ, and η were derived and generalised for the construction of IDF from simulated rainfall. The future IDF projection was obtained through distribution analysis to compute the return period for different durations. The outputs were used to produce a future IDF table consisting of rainfall intensity, duration, and return period. Eq 8 demonstrates the typical IDF relationship for a specific return period obtained from the Department of Irrigation and Drainage Malaysia to calculate the IDF parameters. The calculated parameters are tabulated in Table 4.

**Table 4:** Intensity duration frequency (IDF) curve parameters for all stations (historical IDF). The parameters Station 3216001- Kg Sg Tua, 3217001- Iby Bekalan KM.16, Station 3317001 Air Terjun Sg Batu, and Station 3317004- Genting Sempah.

Rainfall stations/parameters	λ	κ	θ	η	I
**3216001-Kg. Sg. Tua**	68.38	0.15	0.29	0.87	55.00
**3217001-Ibu Bekalan KM. 16**	70.12	0.15	0.24	0.87	58.00
**3317001 -Air Terjun Sg. Batu**	64.53	0.16	0.25	0.81	54.00
**3317004 -Genting Sempah**	60.90	0.16	0.28	0.87	49.00
**Average**	65.98	0.15	0.27	0.85	54.00

#### Future IDF curve for Batu Dam catchment

**Fig 11:**
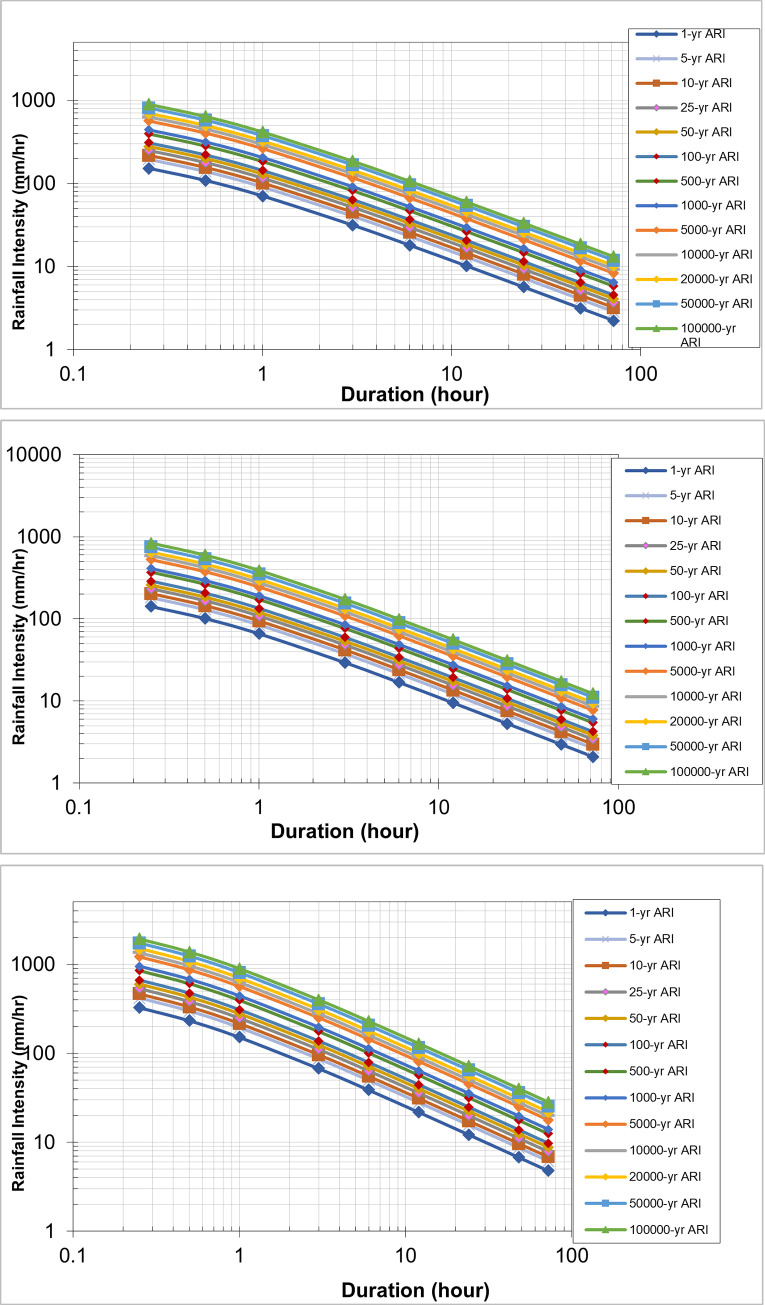
IDF Curve of Early Century under scenario (a) RCP 4.5, (b) RCP 6.0, and (c) RCP 8.5. Rainfall Intensity vs. Duration for Various Average Recurrence Intervals (ARI), illustrating the decrease in intensity with longer storm durations across different ARI events, ranging from 1-year to 100,000-year return periods.

**Fig 12:**
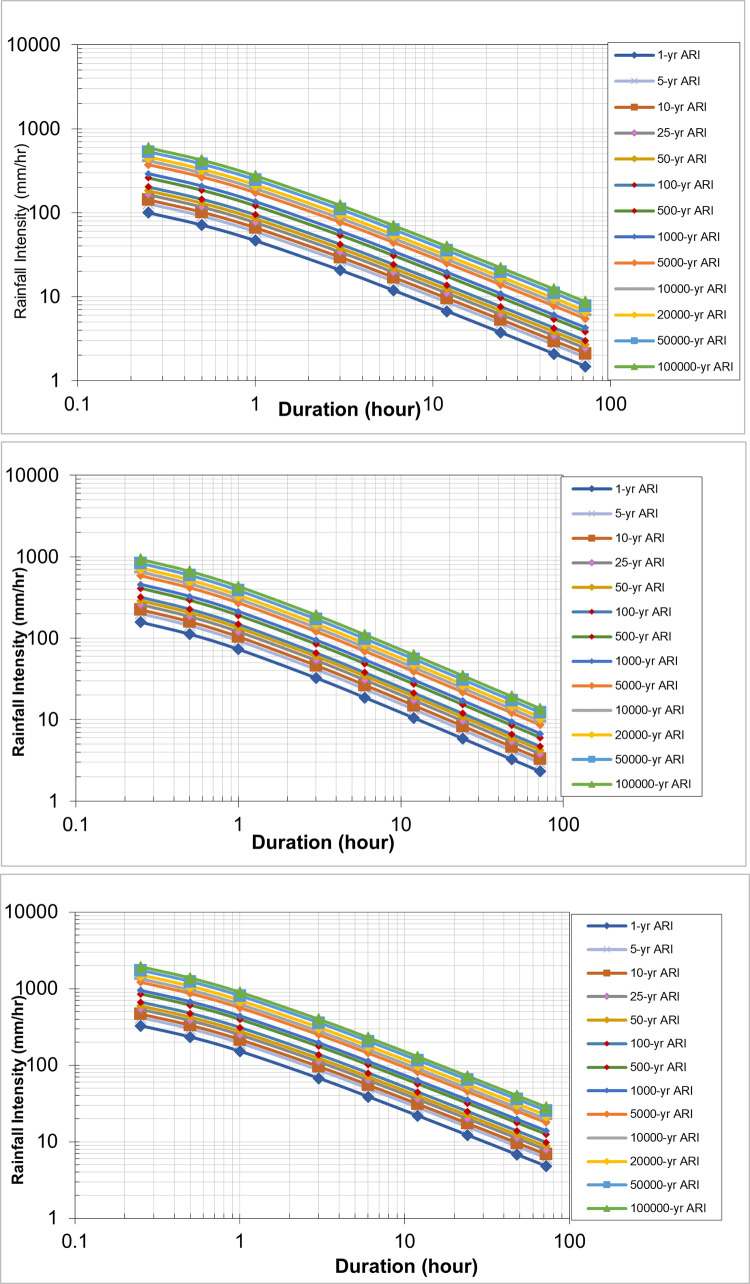
IDF Curve of Mid Century under scenario (a) RCP 4.5, (b) RCP 6.0, and (c) RCP 8.5. Rainfall Intensity vs. Duration for Various Average Recurrence Intervals (ARI), illustrating the decrease in intensity with longer storm durations across different ARI events, ranging from 1-year to 100,000-year return periods.

**Fig 13:**
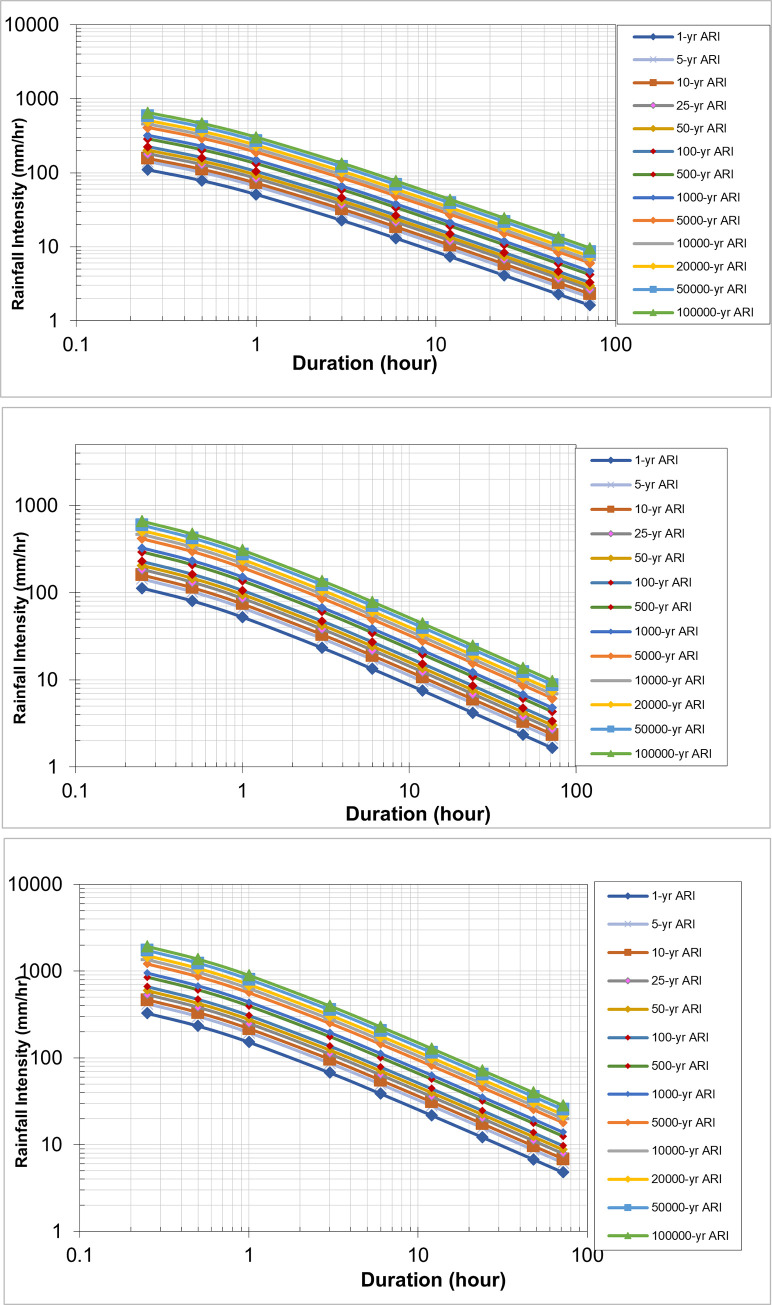
IDF Curve of Late Century under scenario (a) RCP 4.5, (b) RCP 6.0, and (c) RCP 8.5. Rainfall Intensity vs. Duration for Various Average Recurrence Intervals (ARI), illustrating the decrease in intensity with longer storm durations across different ARI events, ranging from 1-year to 100,000-year return periods.

The corrected projected areal rainfall data from the Batu Dam catchment for a 1-hour period were used to generate IDF curves. These curves were generated for a range of time periods, including 5, 10, 20, 50, 100, 500, and 1000 years, as well as 5000, 10,000, 20,000, 50,000, and 100,000 years. Table 5 provides the values of CCF for deriving future IDF curves for different return periods, as shown in Figs 11–13.

**Table 5:** Maximum annual rainfall at 1-hr events and the corresponding CCF (corrected future projection). The table provides the Maximum Annual Rainfall at 1 hour (mm) and Climate Change Factor (CCF) for different stations over three time periods (Early Century, Mid Century, Late Century) and under three climate change scenarios (RCP4.5, RCP6.0, and RCP8.5).

**Station**	**Maximum Annual Rainfall at 1 hr (mm)**								
	**Early Century**			**Mid Century**			**Late Century**		
	**RCP4.5**	**RCP6.0**	**RCP8.5**	**RCP4.5**	**RCP6.0**	**RCP8.5**	**RCP4.5**	**RCP6.0**	**RCP8.5**
3216001	70.38	66.73	153.83	42.33	74.00	153.80	51.00	50.22	153.83
3217001	50.28	53.42	63.98	50.00	49.07	44.68	34.00	56.22	57.04
3317001	70.34	39.22	44.03	42.33	35.90	37.69	51.00	47.58	58.03
3317004	28.39	23.29	28.52	28.25	27.40	31.26	28.00	23.29	34.02
**Station**	**Climate Change Factor (CCF)**								
	**Early Century**			**Mid Century**			**Late Century**		
	**RCP4.5su**	**RCP6.0**	**RCP8.5**	**RCP4.5**	**RCP6.0**	**RCP8.5**	**RCP4.5**	**RCP6.0**	**RCP8.5**
3216001	1.29	1.22	2.81	1.22	1.35	2.81	0.93	0.92	2.81
3217001	0.87	0.92	1.10	0.92	0.85	0.77	0.59	0.97	0.98
3317001	1.31	0.73	0.82	0.73	0.67	0.70	0.95	0.88	1.08
3317004	0.58	0.47	0.58	0.47	0.56	0.64	0.57	0.47	0.69
**Max CCF**	**1.31**	**1.22**	**2.81**	**1.22**	**1.35**	**2.81**	**0.95**	**0.97**	**2.81 **

#### Inflow design flood hydrograph and PMF

**Fig 14:**
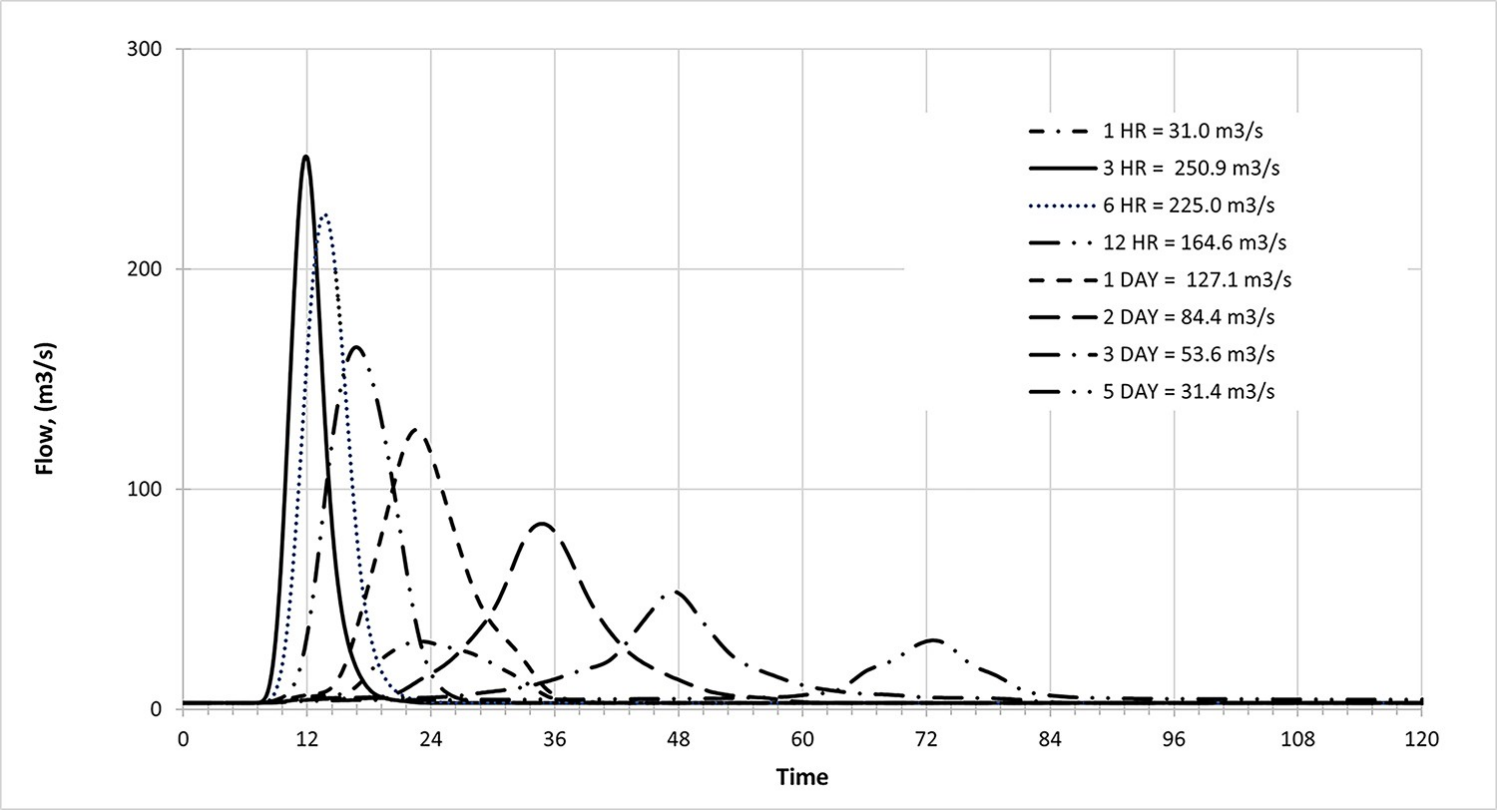
Example PMF simulation of Batu Dam. Flow vs. Time for Various Durations, highlighting peak discharge rates for rainfall events from 1 hour to 5 days.

**Fig 15:**
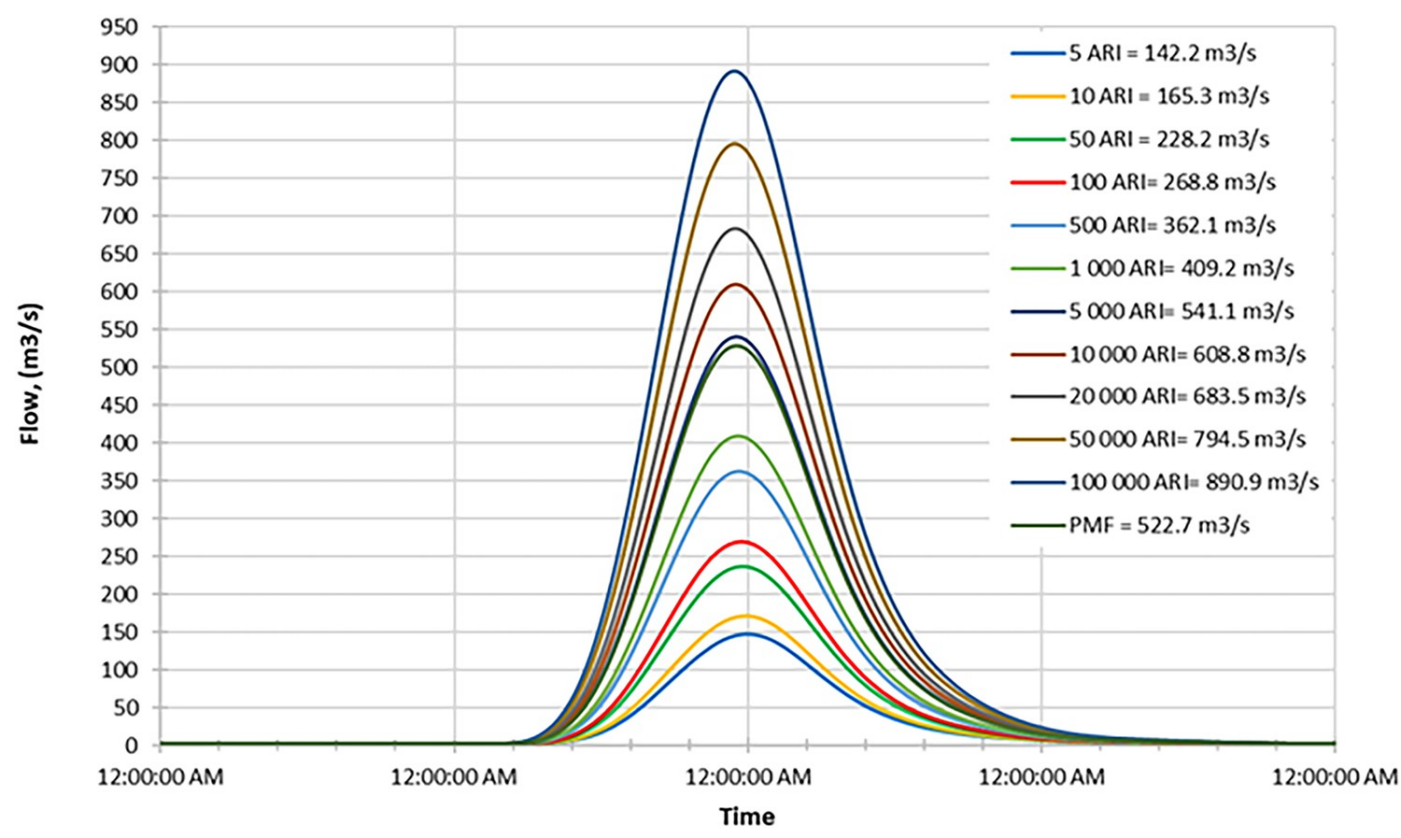
Historical peak inflow design flood at Batu Dam. Flow hydrographs showing peak discharge for ranging from 5 ARI to 100,000 ARI and the Probable Maximum Flood (PMF), illustrating the increase in peak flow with higher ARIs and extreme events.

PMF and IDF for (1, 3, 6, and 12 hours) and 24 hours (1,3 and 5-day) as shown in Fig 14 indicated the extreme flood peak at Batu Dam occurred after three hours with an inflow for the whole century and in different RCP 4.5, 6.0 and 8.5. Fig 15 illustrates the extreme flood peak at Batu Dam occurred after three hours with an inflow for the whole century and in different RCP 4.5, 6.0, and 8.5 scenarios.

#### Historical data hydrograph

The following hydrograph presents historical peak inflow data for Batu Dam under design flood conditions.

### Future data hydrograph

#### Early century

**Fig 16:**
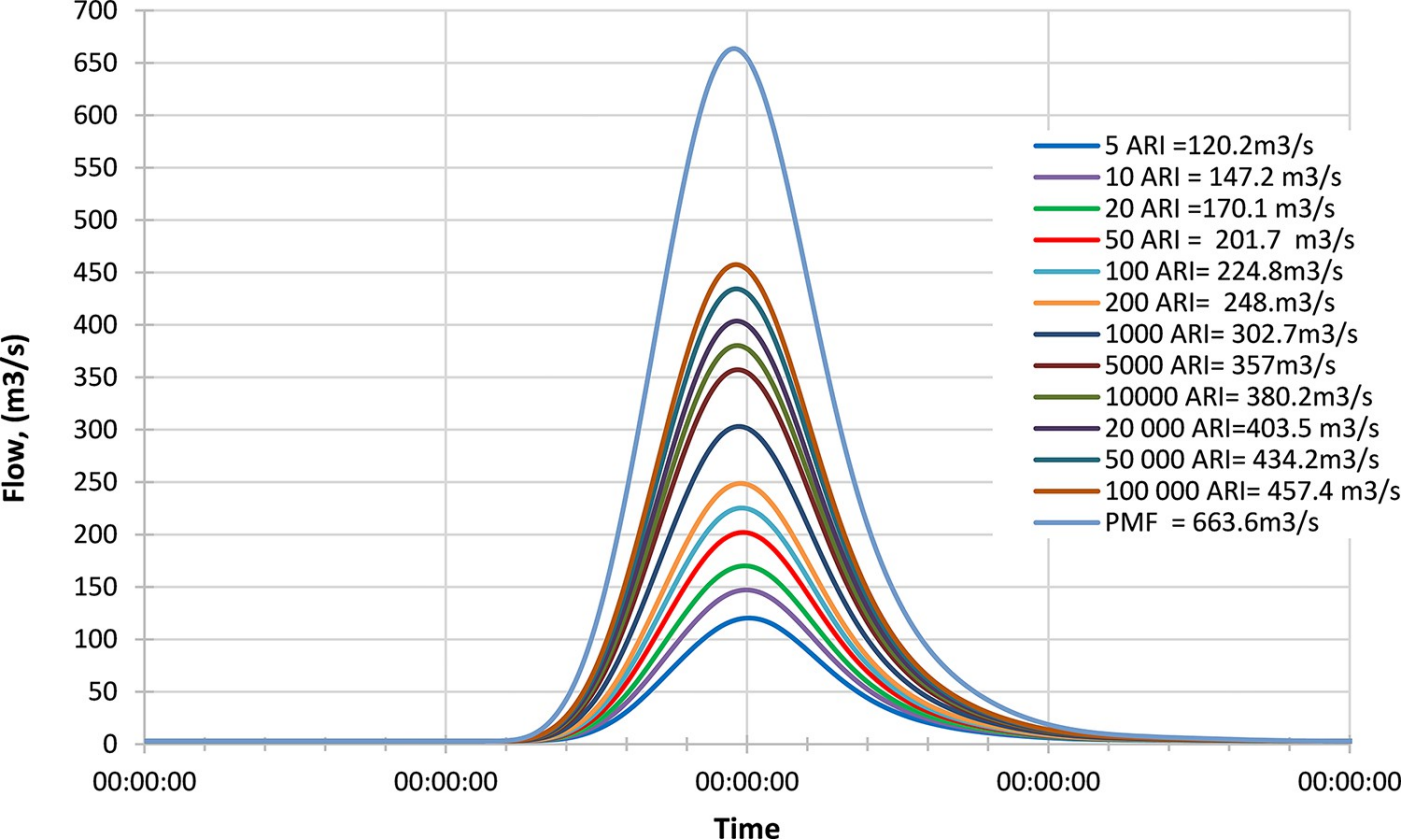
RCP4.5 early century peak inflow design flood at Batu Dam. Flow hydrographs showing peak discharge for ranging from 5 ARI to 100,000 ARI and the Probable Maximum Flood (PMF), illustrating the increase in peak flow with higher ARIs and extreme events.

**Fig 17:**
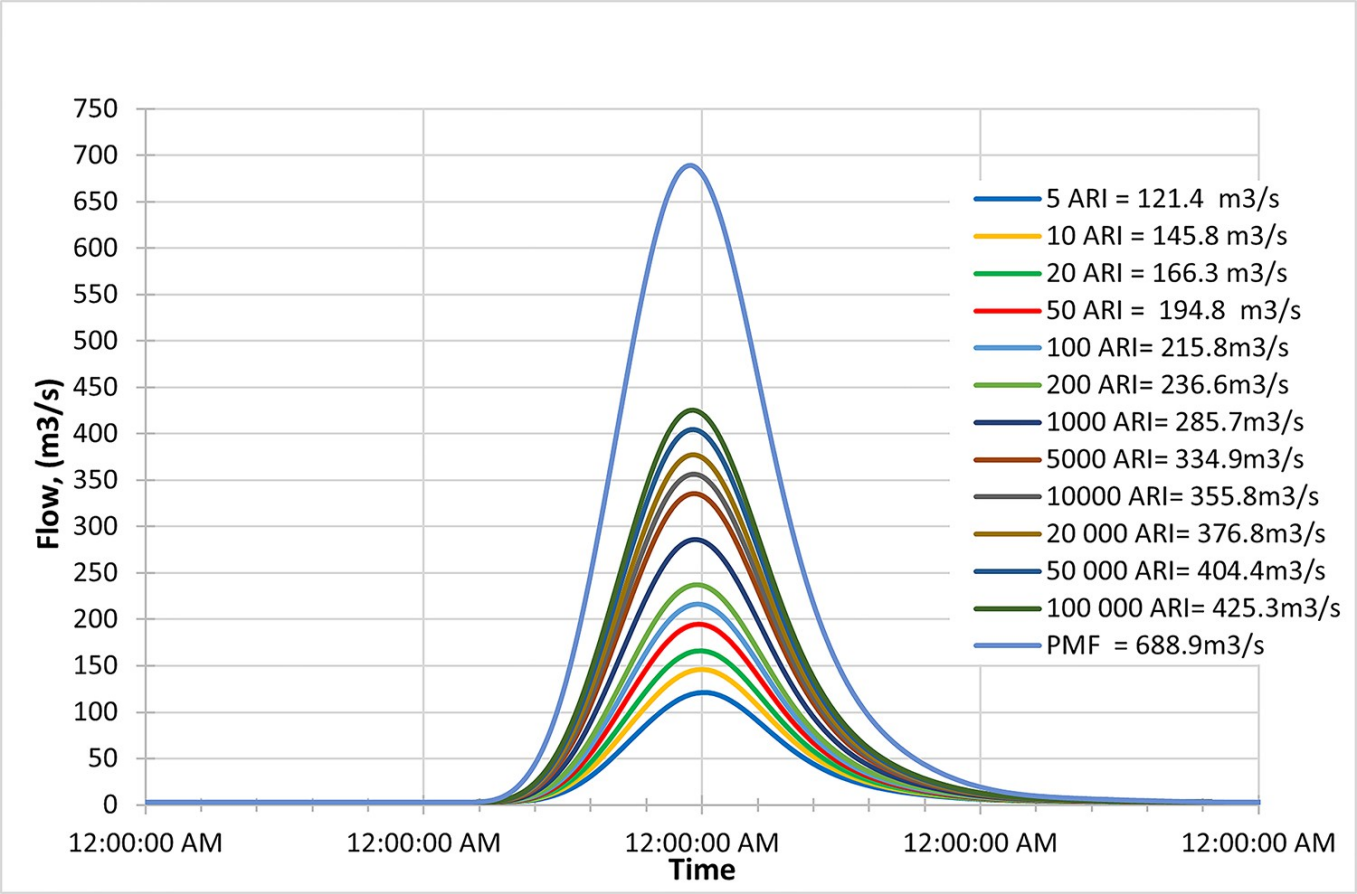
RCP6.0 early century peak inflow design flood at Batu Dam. Flow hydrographs showing peak discharge for ranging from 5 ARI to 100,000 ARI and the Probable Maximum Flood (PMF), illustrating the increase in peak flow with higher ARIs and extreme events.

**Fig 18:**
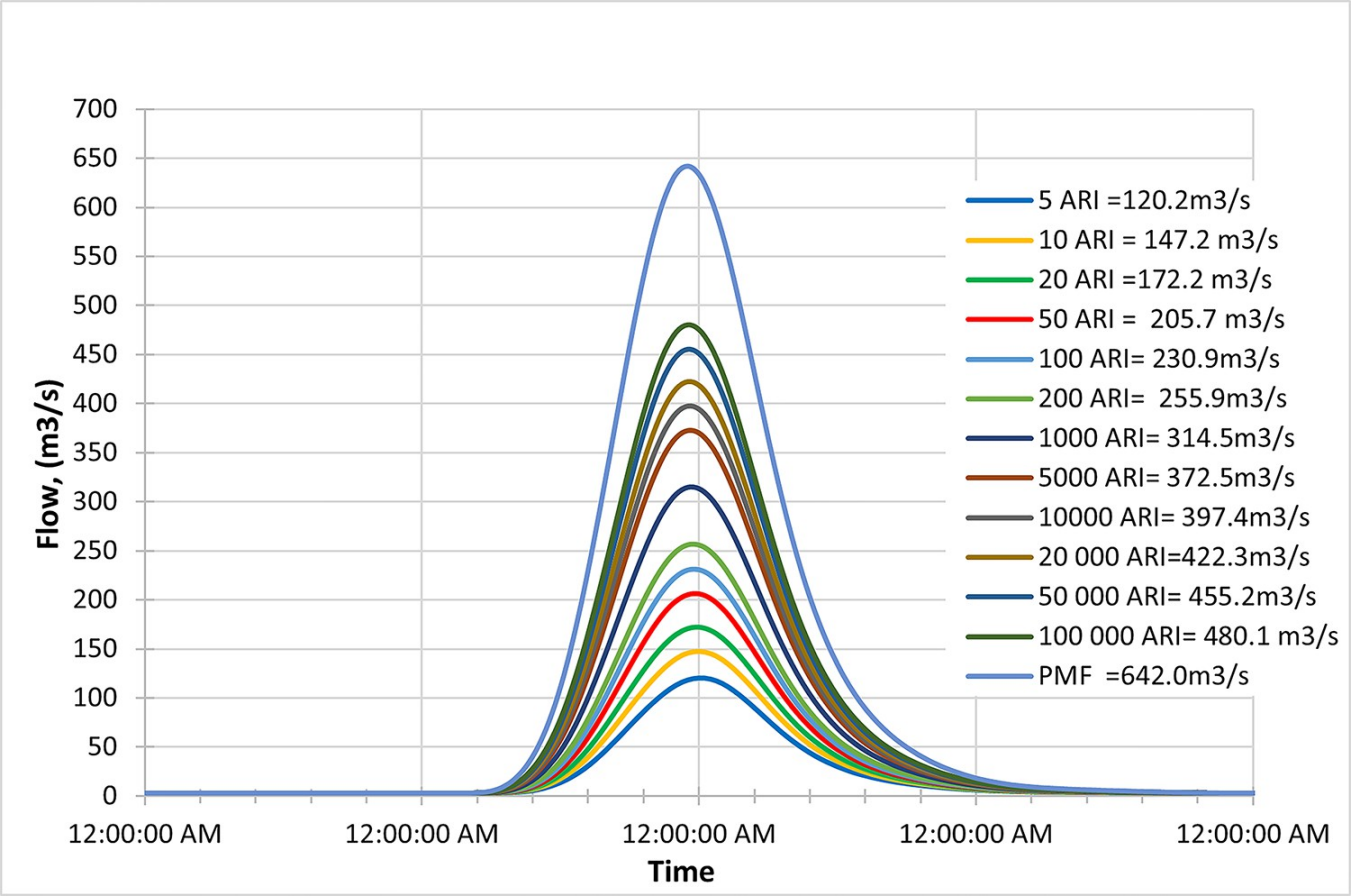
RCP8.5 early century peak inflow design flood at Batu Dam. Flow hydrographs showing peak discharge for ranging from 5 ARI to 100,000 ARI and the Probable Maximum Flood (PMF), illustrating the increase in peak flow with higher ARIs and extreme events.

Figs 16–18 depict the inflow hydrograph for different return periods (ARIs—Average Recurrence Intervals) ranging from 1 year to 100000 years at the beginning of the century. The graph indicates a trend in the inflow design from scenario RCP 4.5 to RCP 8.5. Furthermore, the comparison of the result with the PMF for each scenario reveals the following findings:

- In the RCP 4.5 scenario, the inflow for ARI 50000 (723.3 m^3^/s) is greater than the PMF value in RCP 4.5 (572.2 m^3^/s).
- In the RCP 6.0 scenario, the inflow for ARI 50000 (672.4 m^3^/s) is greater than the PMF value in RCP 6.0 (663.6 m^3^/s).
- In the RCP 8.5 scenario, the inflow for ARI 50 (769.2 m^3^/s) is greater than the PMF value in RCP 8.5 (642.0 m^3^/s).

This information could be crucial for assessing the risk of extreme flooding and designing appropriate infrastructure or flood protection measures. It also suggests that the RCP 8.5 scenario tends to have the highest inflow values for various return periods compared to the other scenarios.

#### Mid century

**Fig 19:**
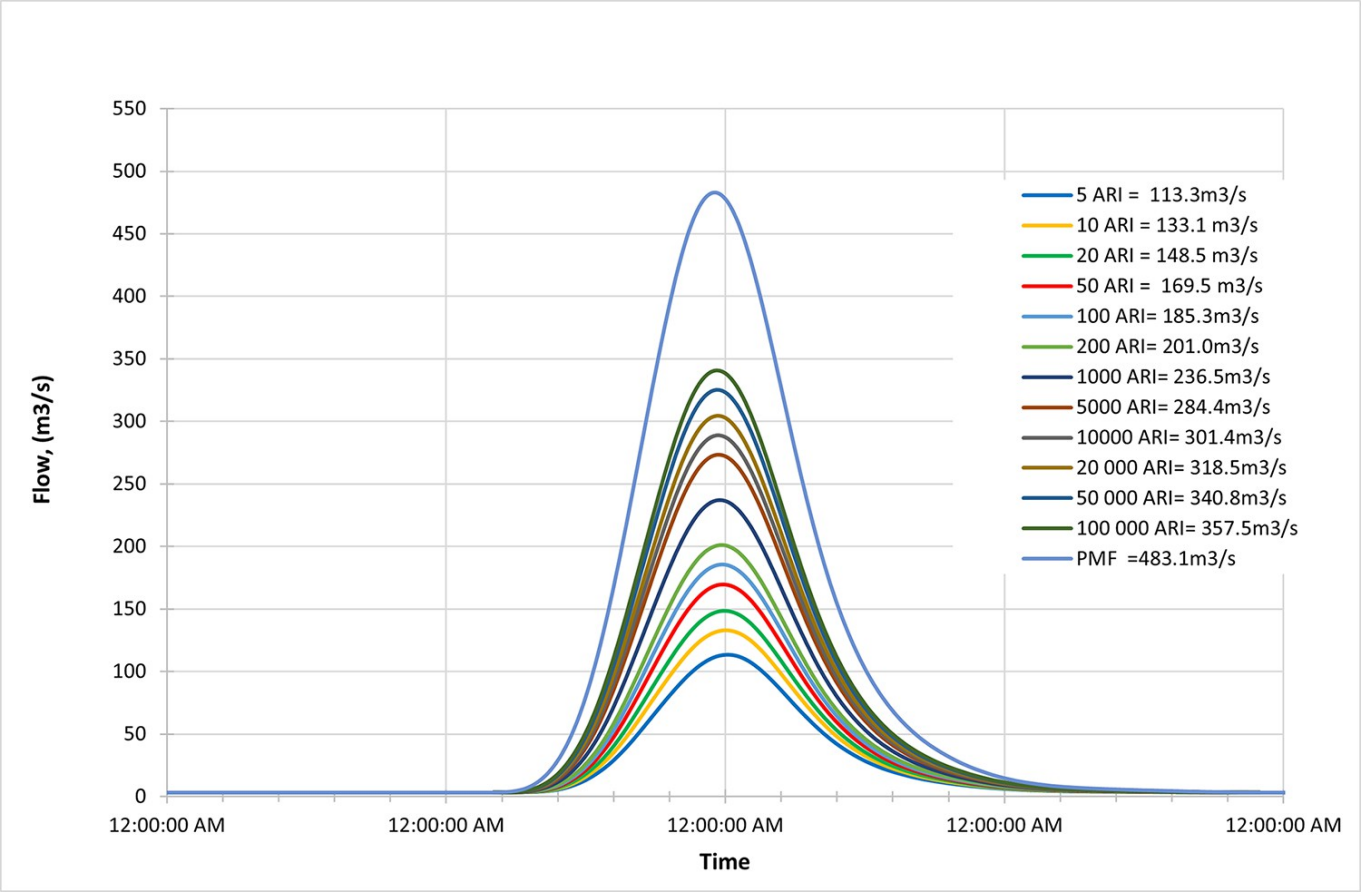
RCP4.5 mid century peak inflow design flood at Batu Dam. Flow hydrographs showing peak discharge for ranging from 5 ARI to 100,000 ARI and the Probable Maximum Flood (PMF), illustrating the increase in peak flow with higher ARIs and extreme events.

**Fig 20:**
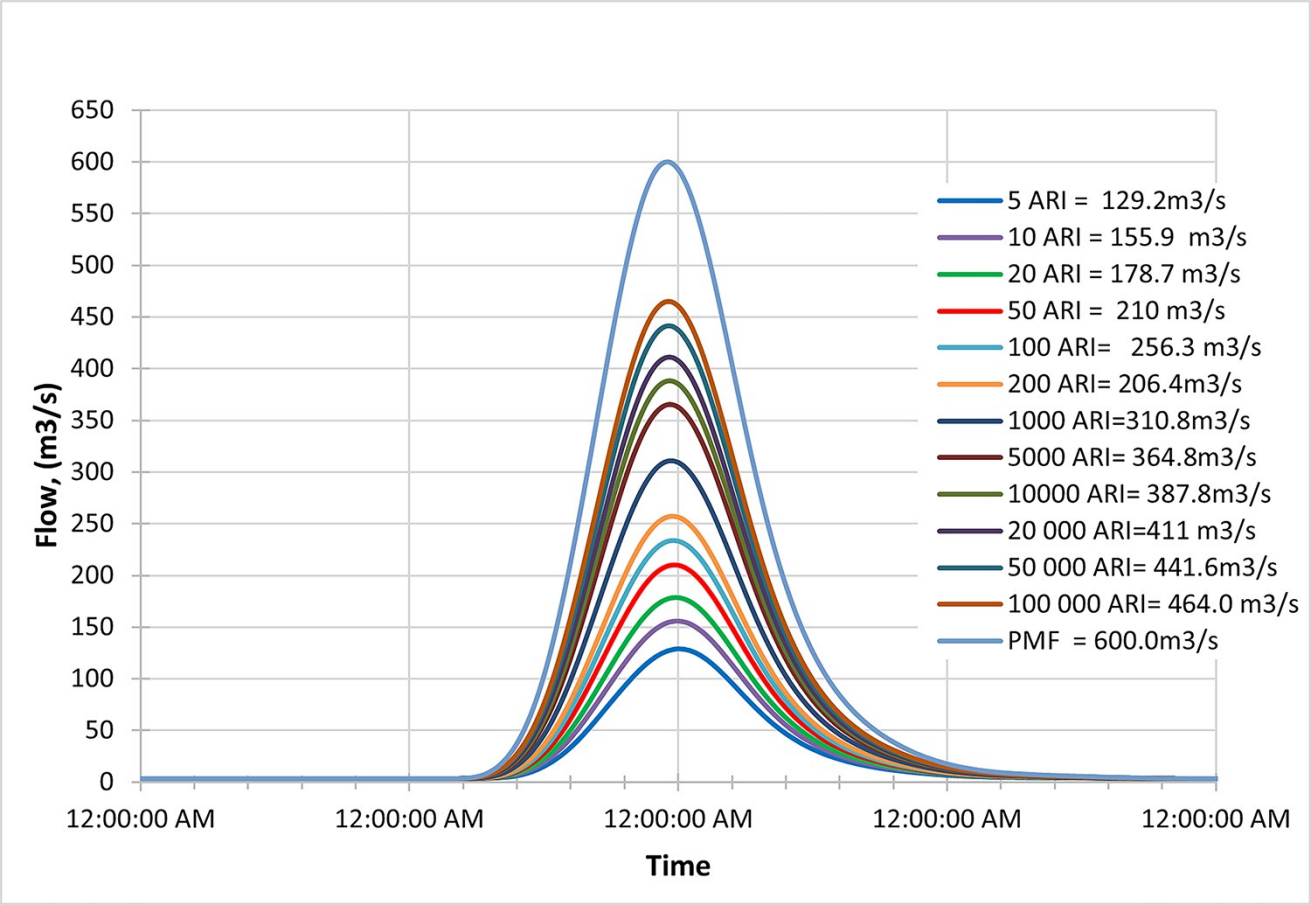
RCP6.0 mid century peak inflow design flood at Batu Dam. Flow hydrographs showing peak discharge for ranging from 5 ARI to 100,000 ARI and the Probable Maximum Flood (PMF), illustrating the increase in peak flow with higher ARIs and extreme events.

**Fig 21:**
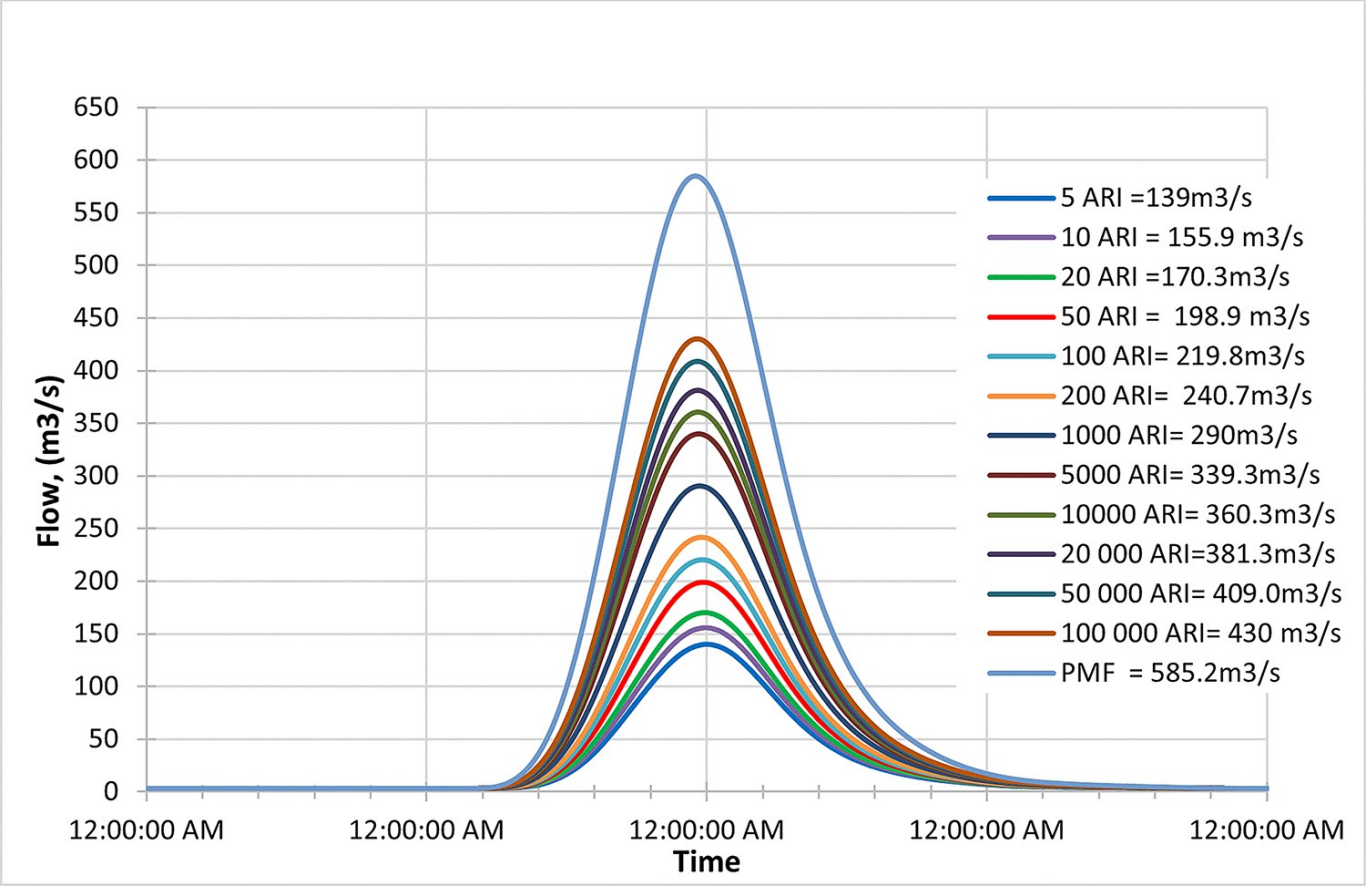
RCP8.5 mid century peak inflow design flood at Batu Dam. Flow hydrographs showing peak discharge for ranging from 5 ARI to 100,000 ARI and the Probable Maximum Flood (PMF), illustrating the increase in peak flow with higher ARIs and extreme events.

Figs 19–21 display the resultant inflow design at the mid-century for different return periods (ARIs) ranging from 1 year to 100000 years. The graph shows a trend in inflow design from scenario RCP 4.5 to RCP 8.5, indicating variations in water inflow over different scenarios. Furthermore, by comparing the results with the PMF for each scenario, we observed the following findings:

- In the RCP 4.5 scenario, the inflow for ARI 50000 is greater than the PMF value in RCP 4.5.
- In the RCP 6.0 scenario, the inflow for ARI 50000 is greater than the PMF value in RCP 6.0.
- In the RCP 8.5 scenario, the inflow for ARI 50 is greater than the PMF value in RCP 8.5.

From these findings, we can deduce that for all three scenarios (RCP 4.5, RCP 6.0, and RCP 8.5), the inflow for ARI 50000 and ARI 50 is higher than the corresponding PMF values. This information is essential for understanding the potential risks of extreme flooding events under different climate change scenarios and designing appropriate flood protection measures for each scenario. The data highlights the significance of considering various climate change projections when evaluating water inflow and planning for resilient infrastructure and flood management strategies. Additionally, the inflow at ARI 50000 and ARI 50 might be more critical than the PMF value for each scenario, indicating the importance of preparing for these specific return periods.

### Late century

**Fig 22:**
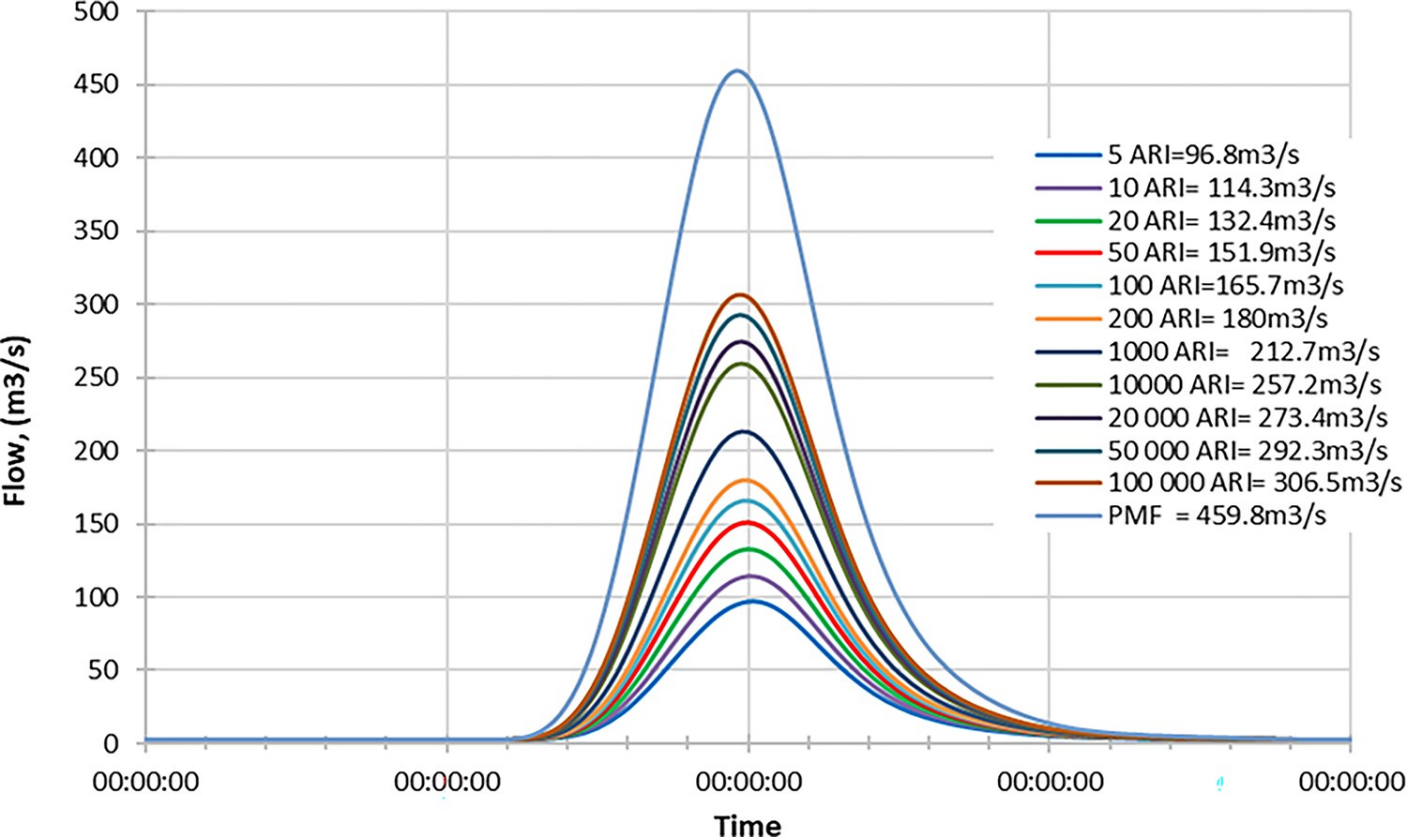
RCP4.5 mid century peak inflow design flood at Batu Dam. Flow hydrographs showing peak discharge for ranging from 5 ARI to 100,000 ARI and the Probable Maximum Flood (PMF), illustrating the increase in peak flow with higher ARIs and extreme events.

**Fig 23:**
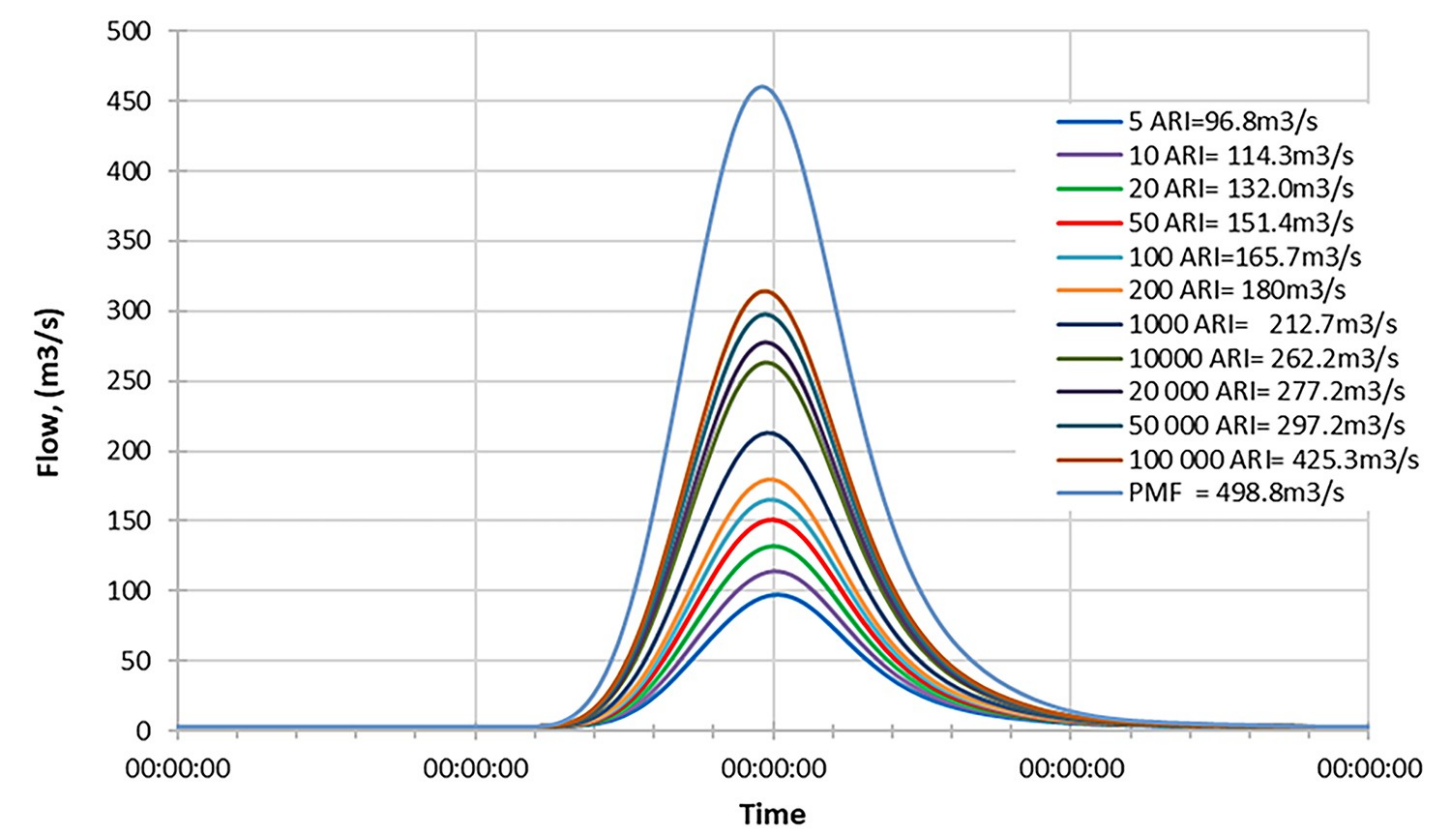
RCP6.0 mid century peak inflow design flood at Batu Dam. Flow hydrographs showing peak discharge for ranging from 5 ARI to 100,000 ARI and the Probable Maximum Flood (PMF), illustrating the increase in peak flow with higher ARIs and extreme events.

**Fig 24:**
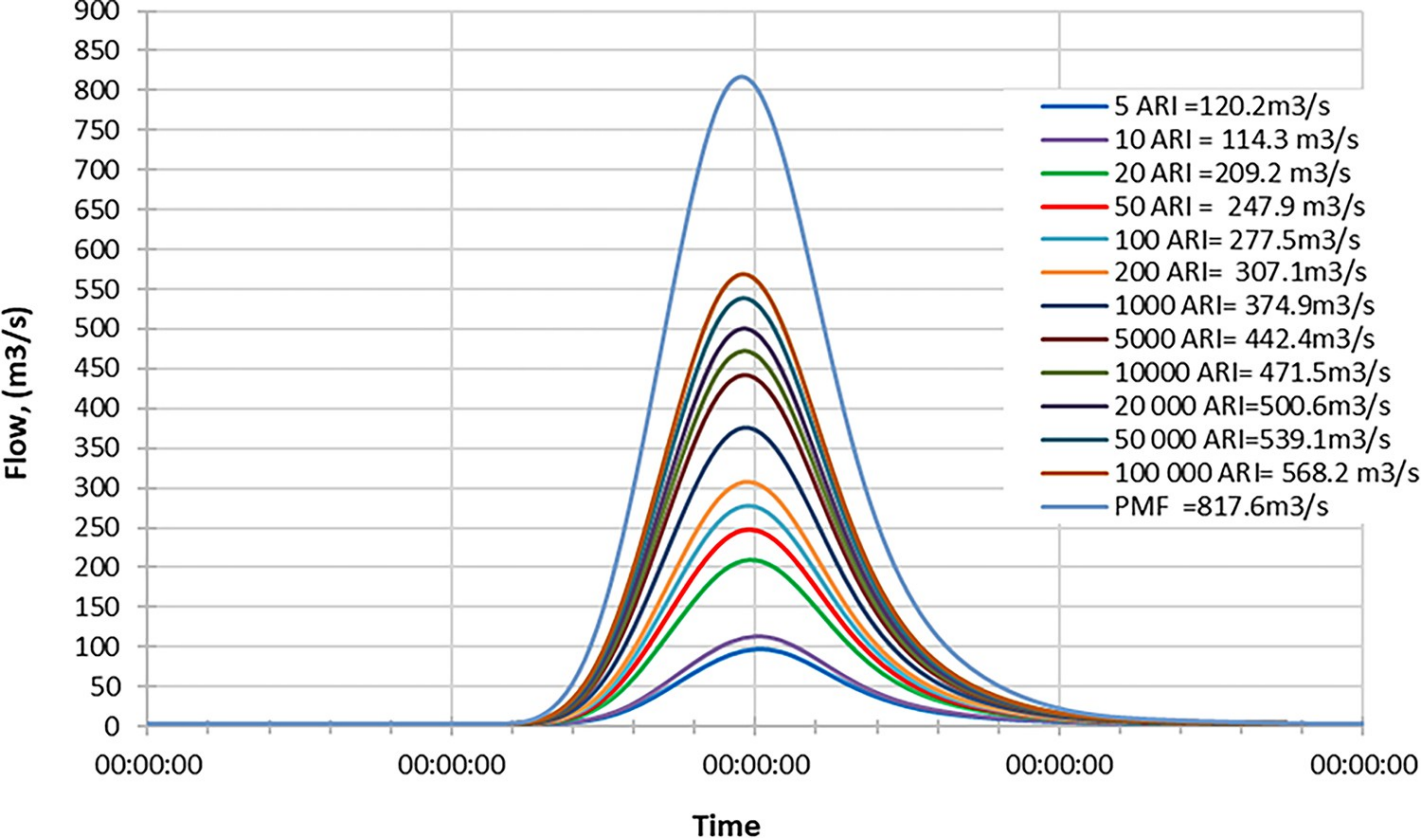
RCP8.5 mid century peak inflow design flood at Batu Dam. Flow hydrographs showing peak discharge for ranging from 5 ARI to 100,000 ARI and the Probable Maximum Flood (PMF), illustrating the increase in peak flow with higher ARIs and extreme events.

Figs 22–24 display the resultant inflow design at the end of the century for different return periods (ARIs) ranging from 1 year to 100000 years. The graph shows a trend in inflow design from scenario RCP 4.5 to RCP 8.5, indicating variations in water inflow over different scenarios.

Furthermore, by comparing the results with the Probable Maximum Flood (PMF) for each scenario, we observe the following findings:

- In the RCP 4.5 scenario, the inflow for ARI 20000 is greater than the PMF value.
- In the RCP 6.0 scenario, the inflow for ARI 50000 is greater than the PMF value.
- In the RCP 8.5 scenario, the inflow for ARI 100 is greater than the PMF value.

From these findings, we can deduce that for all three scenarios (RCP 4.5, RCP 6.0, and RCP 8.5), the inflow for specific return periods is higher than the corresponding PMF values. This information is crucial for understanding the potential risks of extreme flooding events at the end of the century under different climate change scenarios and designing appropriate flood protection measures for each scenario. The data highlights the significance of considering various climate change projections when evaluating water inflow and planning for resilient infrastructure and flood management strategies. Additionally, it suggests that specific return periods such as ARI 20000, ARI 50000, and ARI 100 may pose more significant challenges than the PMF value in their respective scenarios, indicating the importance of preparing for these specific return periods.

These observations are crucial for assessing the potential risks of extreme flooding under different climate change scenarios and designing appropriate flood protection measures for each scenario. The data underscores the significance of considering various climate change projections when evaluating water inflow and planning for resilient infrastructure and flood management strategies.

## Overtopping failure of embankment dam

**Fig 25:**
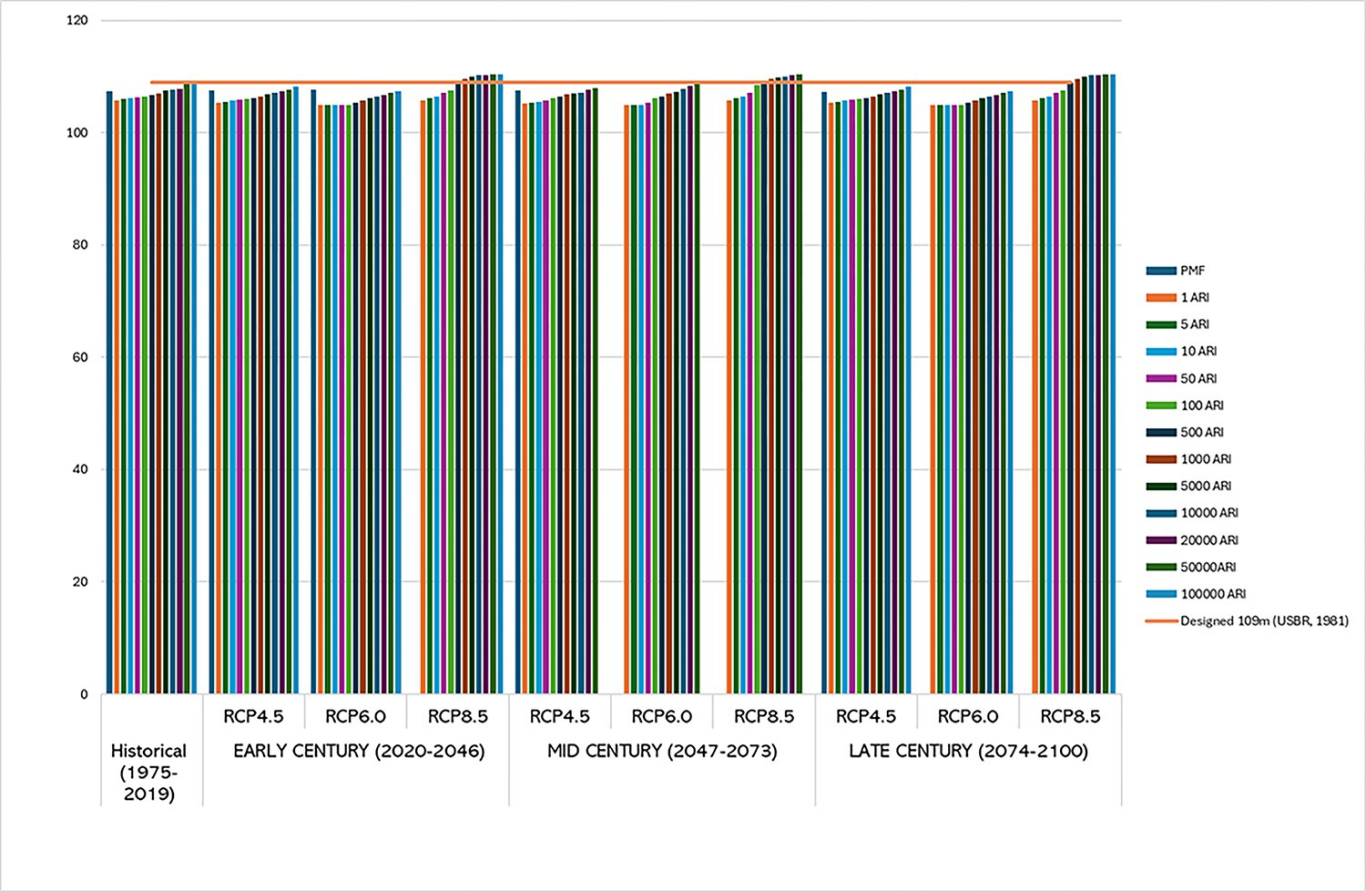
Maximum reservoir level based on inflow for PMF and return period. Projected flood levels between 1 and 100000 Average Recurrence Intervals (ARI) under three emission scenarios: RCP4.5, RCP6.0, and RCP8.5 across different time periods; the historical baseline from 1975–2019 is compared to early-century (2020–2046), mid-century (2047–2073), and late-century (2074–2100) projections.

**Fig 26:**
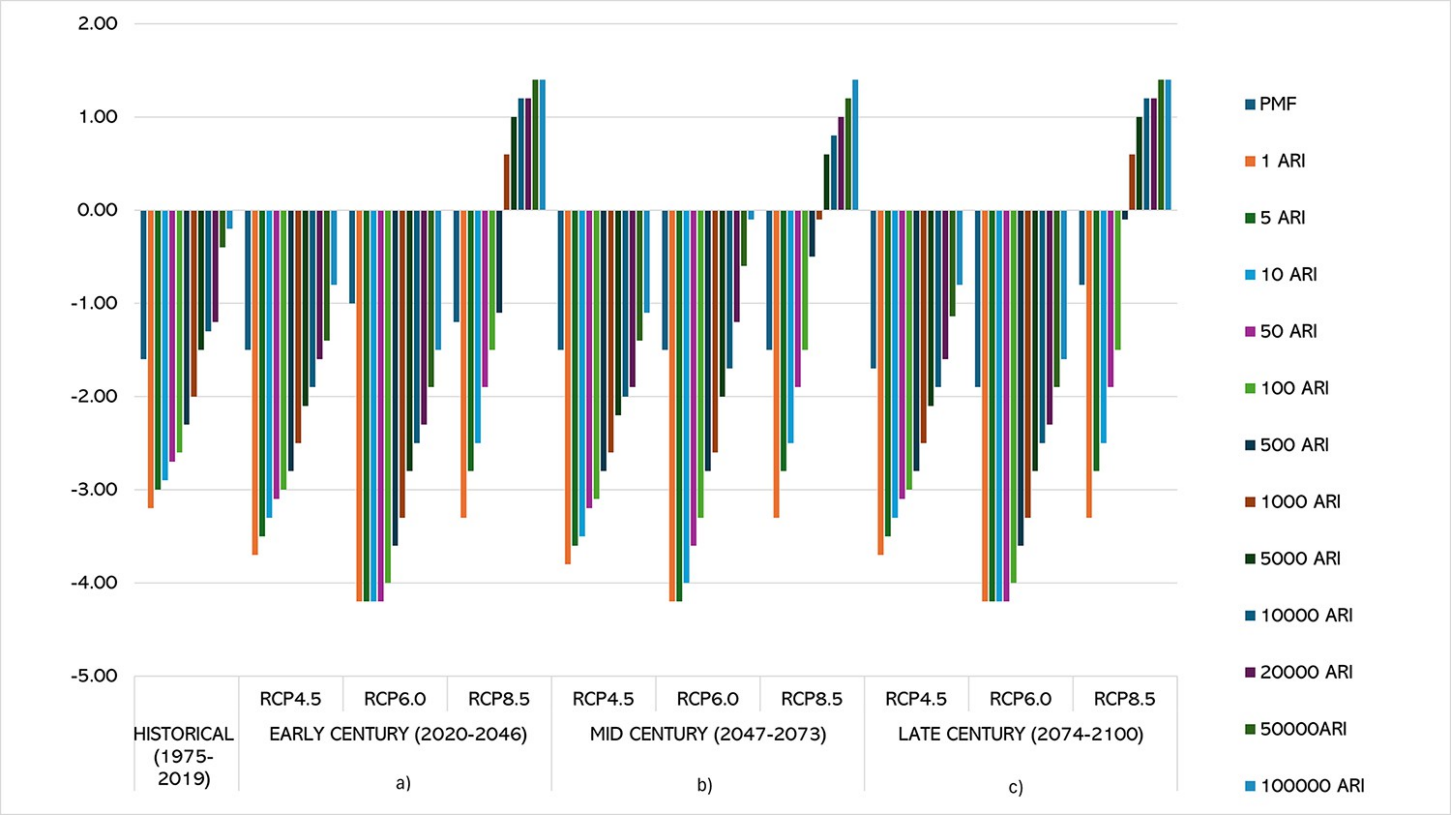
Inflow for PMF and return period between 1 and 100 000-years. hydrograph at (a) Early Century (2020–2046), (b) Mid Century (2047–2073), and (c) Late Century (2074–2100) under different climate scenarios. The upper line indicates inflows exceeding the dam crest level. In the historical period from 1975 to 2019, the water levels have consistently remained below the crest level of embankment dams, indicating no overtopping risk during this timeframe. This observation aligns with the stability and performance of existing dam structures under historical climate conditions. The absence of overtopping incidents suggests that the design and operational protocols in place were effective for the climatic conditions experienced during this period.

Fig 25 shows the maximum water level in the reservoir for historical, Early Century, Mid Century and Late Century compared to designed criteria by USBR in 1981 and previous study of historical data of PMF (Fig 26) by [39] at an initial level 104.85m.

The analysis reveals a clear trend of increasing overtopping risks over time, with the severity magnifying under higher RCP scenarios. The early century presents manageable risks, particularly under RCP 4.5 and 6.0, where adaptive measures can effectively mitigate potential overtopping. However, RCP 8.5 in the early century already shows significant risks that require immediate attention. In the mid-century, the risks become more pronounced, especially under RCP 6.0 and 8.5. The water pool levels nearing or exceeding the crest level indicate the need for proactive measures, including enhancements to spillway capacity and dam reinforcement. The late century period presents the highest risk, with all scenarios showing significant increases in inflow and outflow. The water pool levels under RCP 8.5 consistently exceed the crest level, necessitating comprehensive risk management strategies. Structural reinforcements, increased monitoring, and emergency preparedness plans are critical to mitigate the severe impacts projected.

However, projections for the Early Century (2020–2046) indicate a significant increase in water levels, particularly for higher Average Recurrence Intervals (ARIs). In some scenarios, water levels approach the crest level, suggesting a growing risk of overtopping. This increase can be attributed to the anticipated changes in precipitation patterns and intensities due to climate change. Studies such as those by [35] highlight the need for adaptive strategies to manage these increased risks in the near future.

The Mid Century (2047–2073) projections present a more alarming scenario, with several ARI conditions exceeding the crest level, particularly under RCP 6.0 and RCP 8.5 scenarios. This suggests a tangible overtopping risk that could lead to dam failure if not addressed through structural and operational interventions. [31] emphasize the importance of integrating climate projections into dam safety assessments to preemptively mitigate such risks.

Looking further into the Late Century (2074–2100), the projections show the highest exceedances of the crest level, with multiple ARI values surpassing it. This period is marked by a severe overtopping risk, especially under the RCP 8.5 scenario, which represents the highest greenhouse gas concentration pathway [31, 35]. The findings by [42] reinforce the critical need for robust risk-informed decision-making frameworks to enhance the resilience of embankment dams against the increasingly severe impacts of climate change.

These projections underscore the urgency for proactive measures, including raising crest levels, enhancing spillway capacities, and adopting comprehensive risk management strategies to ensure the continued safety and functionality of embankment dams in the face of escalating climatic threats The findings from [31, 35] support these trends, indicating that climate change substantially increases the likelihood of dam overtopping and failure, especially under the high emissions RCP 8.5 scenario.

## Discussion and conclusions

This study underscores the significant implications of climate change on the safety of embankment dams, particularly highlighting the increasing risk of overtopping under various climate scenarios. The analysis of historical data (1975–2019) and projections for the early, mid, and late 21st century under RCP 4.5, 6.0, and 8.5 scenarios reveals a clear trend of rising water levels and heightened overtopping risks. This study underscores the importance of integrating climate change projections into dam safety management.

The findings highlight the need for immediate and long-term adaptive strategies to address the increasing risks posed by higher inflows and potential overtopping. Ensuring the safety and resilience of embankment dams under future climate scenarios will require a combination of structural enhancements, rigorous monitoring, and adaptive management practices. Consequently, the dam owner must remain vigilant and prepared for future inflow events. Utilizing the future prediction inflow return period can serve as a basis for determining dam decision-making strategies to reduce the impact of discharge on the surrounding community. Ensuring the dam’s safety and resilience against changing climate scenarios is paramount to safeguarding the dam infrastructure and the people living downstream.

Addressing this challenge requires a multi-faceted approach that integrates climate adaptation strategies, infrastructure resilience measures, and robust emergency preparedness protocols. By implementing proactive measures, monitoring systems, and structural upgrades, stakeholders can effectively mitigate the risk of dam overtopping and enhance the safety of communities and ecosystems downstream. Collaboration between government agencies, engineering professionals, and local communities is essential to navigate the complex dynamics of climate change and ensure the sustainable management of embankment dam at Batu Dam, in Malaysia.
